# Distinct prokaryotic and eukaryotic communities and networks in two agricultural fields of central Japan with different histories of maize–cabbage rotation

**DOI:** 10.1038/s41598-023-42291-y

**Published:** 2023-09-18

**Authors:** Harutaro Kenmotsu, Tomoro Masuma, Junya Murakami, Yuu Hirose, Toshihiko Eki

**Affiliations:** 1 Department of Applied Chemistry and Life Science, Toyohashi University of Technology, 1-1 Hibarigaoka, Tempaku, Toyohashi, Aichi 441-8580 Japan; 2 Research Center for Agrotechnology and Biotechnology, Toyohashi University of Technology, 1-1 Hibarigaoka, Tempaku, Toyohashi, Aichi 441-8580 Japan

**Keywords:** Agroecology, Biodiversity, Microbial communities

## Abstract

Crop rotation is an important agricultural practice for homeostatic crop cultivation. Here, we applied high-throughput sequencing of ribosomal RNA gene amplicons to investigate soil biota in two fields of central Japan with different histories of maize–cabbage rotation. We identified 3086 eukaryotic and 17,069 prokaryotic sequence variants (SVs) from soil samples from two fields rotating two crops at three different growth stages. The eukaryotic and prokaryotic communities in the four sample groups of two crops and two fields were clearly distinguished using β-diversity analysis. Redundancy analysis showed the relationships of the communities in the fields to pH and nutrient, humus, and/or water content. The complexity of eukaryotic and prokaryotic networks was apparently higher in the cabbage-cultivated soils than those in the maize-cultivated soils. The node SVs (nSVs) of the networks were mainly derived from two eukaryotic phyla: Ascomycota and Cercozoa, and four prokaryotic phyla: Pseudomonadota, Acidobacteriota, Actinomycetota, and Gemmatimonadota. The networks were complexed by cropping from maize to cabbage, suggesting the formation of a flexible network under crop rotation. Ten out of the 16 eukaryotic nSVs were specifically found in the cabbage-cultivated soils were derived from protists, indicating the potential contribution of protists to the formation of complex eukaryotic networks.

## Introduction

Soils are complex and invaluable terrestrial media. Soil organisms, such as bacteria, fungi, or protists, play crucial roles in nutrient cycles in terrestrial ecosystems^1^ and their communities are influenced by soil environments and plants. In particular, the potential interactions of soil organisms with crops cultivated in agricultural fields can affect the growth and health of crops via microorganism-derived nutrients and plant pathogens^2,3^. Therefore, quantitative data of prokaryotic and eukaryotic communities and their taxonomic changes during crop cultivation are useful for monitoring and accessing the biological environment of agricultural soils. High-throughput sequencing of amplicons derived from the 16S ribosomal RNA (rRNA) gene and 18S rRNA gene cluster (i.e., DNA metabarcoding) is a powerful tool for analyzing soil prokaryotic and eukaryotic communities (mainly bacteria and fungi) in agricultural soils because of the crucial roles of these organisms in the pedosphere^4^. Previous DNA metabarcodings clarified the taxonomic compositions of major soil organisms such as bacteria, fungi, and protists in agricultural areas as well as their changes with fertility, tillage, and other types of agricultural practices. Crop rotation has been widely used in crop cultivation to suppress plant diseases and replant failure by changing crops in cultivation cycles to avoid continuous cropping^3^. Several studies using DNA metabarcoding have been reported for soil organisms living in agricultural fields under crop rotation with different crops and cropping sequences, cycles, and periods^5–33^ and with different agricultural management systems, such as tillage and fertility. However, most of these studies were focused on soil bacteria and fungi and details of whole soil organisms, especially nonfungal eukaryotes, are poorly understood in crop-rotation fields. We assumed that crop rotation could also influence soil nonfungal eukaryotes, and investigated both the eukaryotic and prokaryotic communities in the field soils under crop rotation in this study, to verify the hypothesis.

We previously applied 18S rRNA gene-derived amplicon sequencing using Illumina MiSeq to analyze soil nematodes and successfully clarified the nematode communities in sweet potato-cultivated fields^34^. Herein, we applied DNA metabarcoding to investigate both prokaryotes and eukaryotes living in two fields (i.e., field_1 and field_2) cropping maize and cabbage by rotation in central Japan in 2019. The maize–cabbage rotation was performed to prevent clubroot diseases of cabbage^35^ in both fields in this year, although each field had a different history of agricultural management in the previous year: field_1 was managed as fallow and was treated with green manure, and field_2 underwent the maize–cabbage rotation in 2018. Thirty-six sample soils from two fields cultivating two crops at three different growth stages were analyzed, and unique sequence variants (SVs) of the 16S and 18S rRNA genes were identified. We then investigated the prokaryotic and eukaryotic taxonomic compositions of the SVs, analyzed the α- and β-diversities of the communities in the samples, and assessed the sample-soil chemical parameter relationships via redundancy analysis (RDA). Finally, we characterized the networks of prokaryotic and eukaryotic SVs in four sample groups (i.e., maize- and cabbage-cropping soils in two fields). Using these analyses, we clarified the biological features of two agricultural fields cropping maize and cabbage by rotation with different management history.

## Results

### Eukaryotic and prokaryotic taxonomic compositions in field soils under maize–cabbage rotation

**Figure 1:**
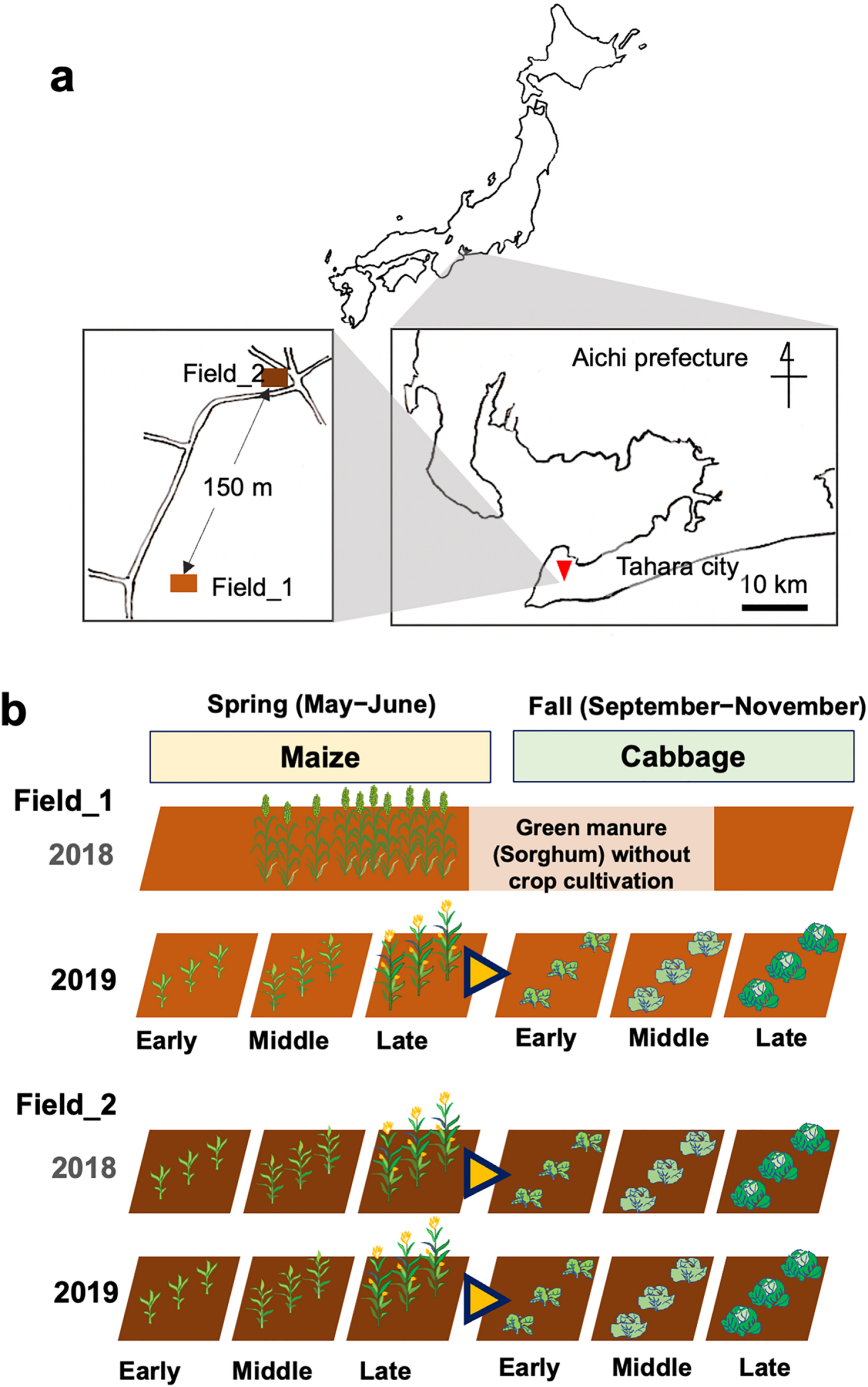
Experimental sites (**a**) and scheme (**b**) of soil sampling in this study. Soil sampling was performed in the closely located two fields (i.e., field_1 and field_2) with different rotation history in Tahara city in central Japan (**a**), where maize and cabbage were cultivated between May to June and September to November in 2019, as shown in (**b**). In 2018, field_1 remained without crop cultivation and was treated with green manure (sorgham), and the first maize–cabbage rotation cycle was performed in field_2. Soils were isolated from three sampling sites near the crops at the early, middle, and late stages of plant growth. Yellow triangles surrounded by blue line: before cabbage cultivation, the fields were tilled with maize residues, supplied by fertilizer, and treated with flusulfamide to prevent clubroot disease.

**Figure 2:**
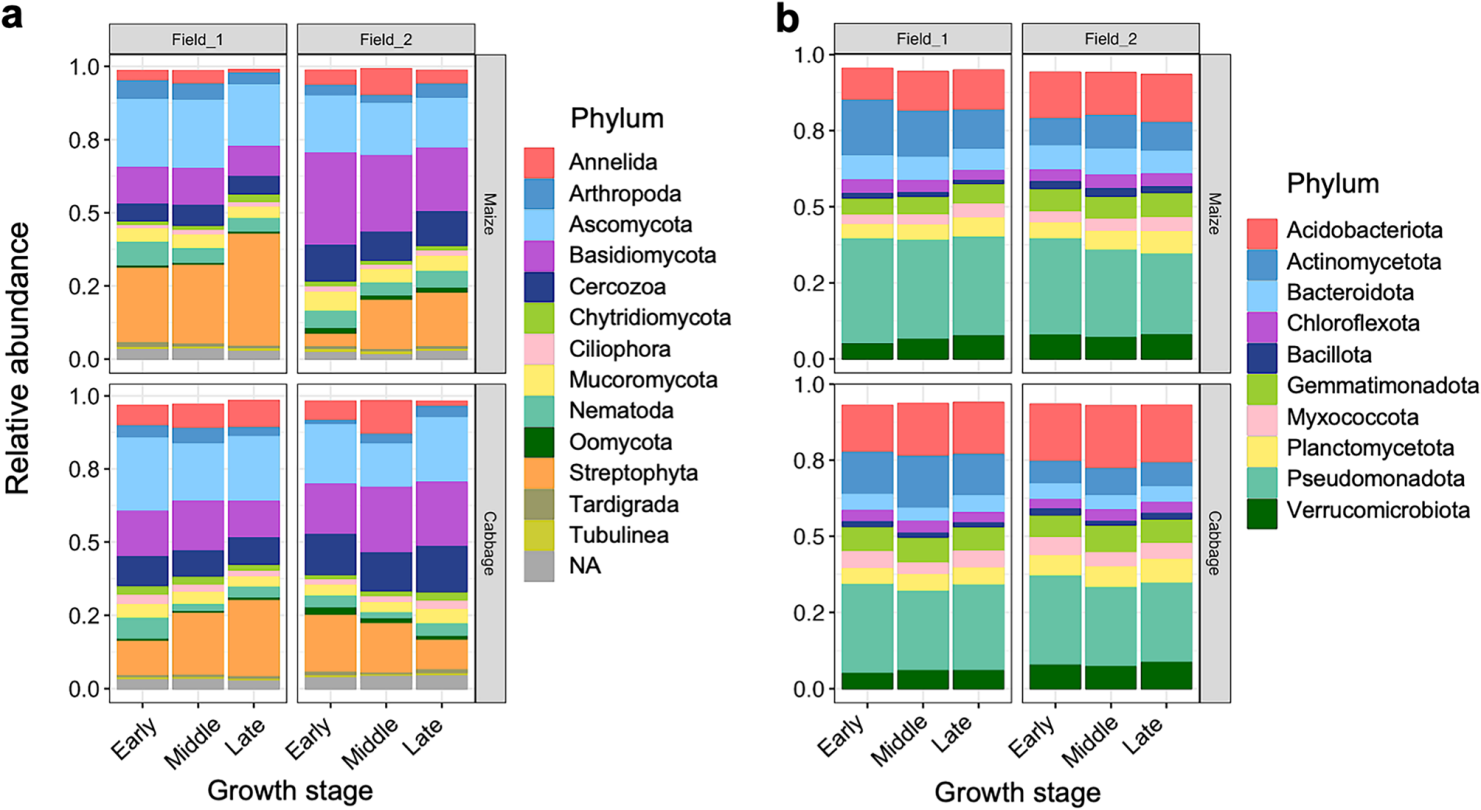
Relative abundance of eukaryotic (**a**) and prokaryotic (**b**) phyla in the soils from two agricultural fields cultivating maize and cabbage at three different growth stages. Phyla are indicated by colors as shown in the right of figures. *NA* not assigned phylum.

Soils were sampled from the three independent sites near plants in field_1 and field_2 cultivated with maize and subsequently with cabbage in 2019 at three different growth stages (i.e., early, middle, and late). Field_1 was fallow without cropping and was treated with green manure in 2018, and the maize–cabbage rotation was performed in field_2 in the year (Fig. 1). Sequencing of 18S and 16S rRNA gene-derived amplicons from DNA isolated from 36 soil samples identified 3086 and 17,069 SVs, respectively, and their taxa were assigned according to the SILVA database. Eukaryotic SVs were dominantly derived from the Ascomycota, Basidiomycota, and Cercozoa phyla, indicating a high abundance of fungi in the field soils (Fig. 2a). The fractions of the Streptophyta phylum were also dominant but were only derived from a single SV from the cultivated maize (SV_1) and cabbage (SV_3). The top three phyla of prokaryotic SVs were Pseudomonadota, Acidobacteriota, and Actinomycetota, followed in abundance by the Bacteroidota, Gemmatimonadota, Planctomycetota, Verrucomicrobiota, Chloroflexota, Myxococcota, and Bacillota (Fig. 2b). Regarding the family- and genus-level compositions, five major eukaryotic families (Mrakiaceae, Mortierellaceae, Filobasidiaceae, Chaetomiaceae, and Euglyphida [see the Methods section]) and several prokaryotic families and genera were identified, some of which were differently abundant according to field and crop; moreover, their relative abundances changed under cabbage cultivation (Supplementary Fig. S1 and Table S1). For instance, the fungal family Mrakiaceae, the prokaryotic family Sphingomonadaceae, and the genus *Sphingomonas* were more abundant in the field_2 soils vs. the field_1 soils, and their relative abundances were decreased by cabbage cultivation. In addition, we investigated the relative abundance of an SV for *Plasmodiophora brassicae* (SV_97), which causes clubroot diseases of cabbage^35^, in the soils, and successfully detected SV_97 in the maize-cultivated field_2 soils. The abundance of SV_97 decreased with crop growth (Supplementary Fig. S2).

### The α- and β-diversities in the maize- and cabbage-cultivated field soils

**Figure 3:**
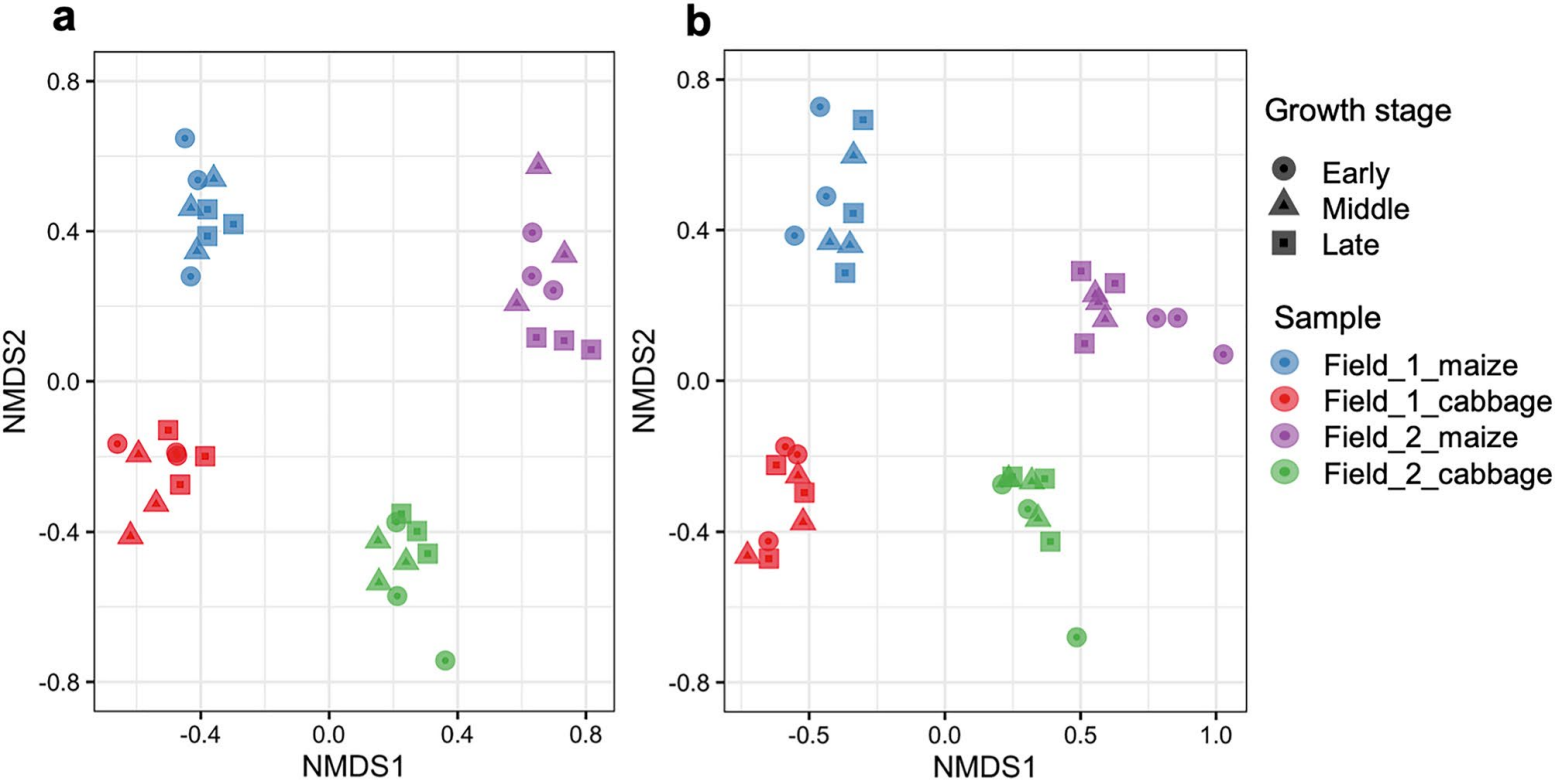
Nonmetric multidimensional scaling (NMDS) ordinations showing differences in β-diversity, based on Bray–Curtis dissimilarity among eukaryotic (**a**) and prokaryotic (**b**) communities in the samples. Three samples were derived from field_1 cropping maize (blue) and cabbage (red) and field_2 cropping maize (purple) and cabbage (green). The early, middle, and late stages of plant growth are indicated by circles, triangles, and squares, respectively.

We determined the α- and β-diversities to access the diversities of eukaryotic and prokaryotic communities in the maize- and cabbage-cultivated field soils. The Shannon indexes of eukaryotic SVs for α-diversity increased with crop rotation from maize to cabbage in both fields (Supplementary Fig. S3a). Especially, the significant increase of the Shannon indexes was found in field_1 soils and the eukaryotic indexes were comparable in both cabbage-cultivated fields (Supplementary Table S2); however, those of the prokaryotic SVs slightly decreased in field_1 soils but did not change in field_2 soils (Supplementary Fig. S3b). In addition, the α-diversities of eukaryotic SVs in field_1 decreased with the growth of both crops but not in field_2 (Supplementary Fig. S3c). The Shannon indexes of prokaryotic SVs in the maize-cultivated field soils tended to increase with crop growth but not in cabbage-cultivated field soils (Supplementary Fig. S3d). The β-diversities were determined based on the Bray–Curtis distances to investigate the difference in soil communities of eukaryotes and prokaryotes among the samples. Four sample groups (i.e., maize- and cabbage-cultivated soil samples from field_1 and field_2) were significantly separated in the resultant nonmetric multidimensional scaling (NMDS) plots of eukaryotic (Fig. 3a) and prokaryotic SVs (Fig. 3b) (adonis2 test: *R*^2^ = 0.66 and *P* = 0.001 and *R*^2^ = 0.51 and *P* = 0.001, respectively), suggesting the presence of distinct communities between the maize- and cabbage-cultivated soils as well as in the two field soils. The prokaryotic diversities of three samples isolated from two fields cropping maize at the early stage mapped differently from those of corresponding samples at the middle and late stages (Fig. 3b).

### Clustering and RDA of soil chemical parameters

**Figure 4:**
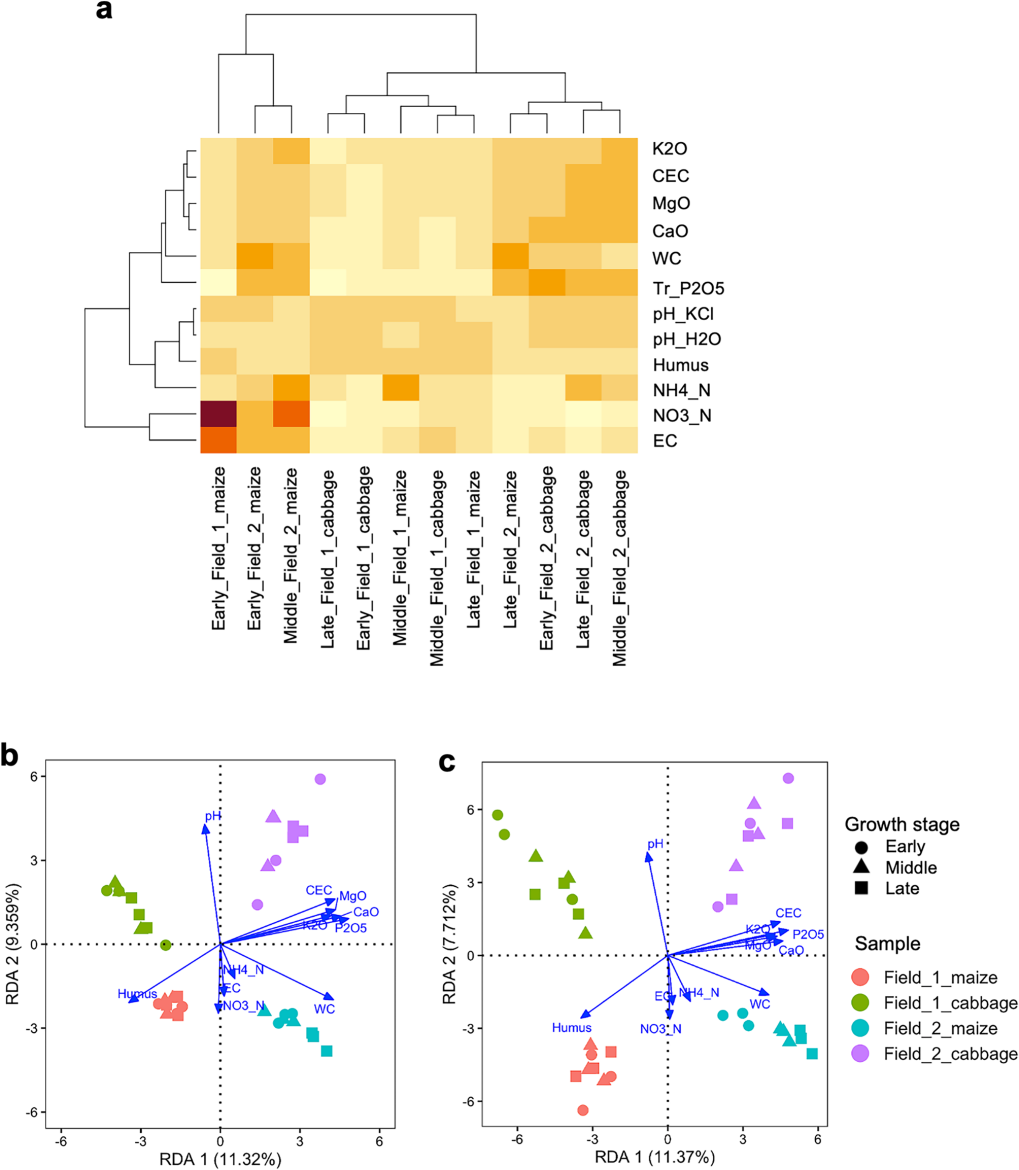
(**a**) Heatmap of soil chemical parameters in the samples. Samples are clustered by the relative contents of chemical parameters. Abbreviations of chemical properties: cation-exchange capacity (CEC), pH in water and KCl solution (pH_H2O, pH_KCl), nitrate nitrogen (NO3_N), ammonium nitrogen (NH4_N), exchangeable potassium (K2O), exchangeable magnesium (MgO), exchangeable calcium (CaO), electric conductivity (EC), available phosphorus (Tr_P2O5), humus content (Humus), and water content (WC). The raw data are shown in Supplementary Table S3. Redundancy analysis (RDA) of eukaryotic (**b**) and prokaryotic (**c**) communities in the samples showing soil chemical properties as vectors. The colors and symbols for sample groups and growth stages are shown in the legend for Fig. 3. Longer arrows indicate a stronger association.

To assess the chemical properties of 12 sample soils (two fields, two crops, and three growth stages), 12 parameters of soils were measured (Supplementary Table S3). The resultant heatmap indicated two clusters for the sample soils: one cluster contained three samples from the fields cultivated with maize at the early and middle growth stages (Fig. 4a), and these soils had a high nitrate and ammonium nitrogen content and high electric conductivity, which are probably caused by fertilizer supply before maize cultivation. The field_2-derived soils contained a higher content of nutrients such as potassium, calcium, magnesium, and phosphates and of water. The field_1-derived soils contained the highest content of humus, which were likely derived from green manure applied in the previous year. RDA was performed to investigate the relationship between the soil parameters and the SVs in the sample soils. The resultant plots of eukaryotic and prokaryotic SVs were comparable, and the following relationships were detected as shown in (Fig. 4b,c): field_1-derived SVs were related to humus content; field_2-derived SVs were related to water content, three exchangeable ions and available phosphorus content, and cation-exchange capacity; the SVs derived from the maize-cultivated field soils were related to ammonium and nitrate nitrogen contents, humus content, electric conductivity, and water content; and the SVs derived from the cabbage-cultivated field soils were related to pH. In addition, the SVs derived from the maize-cultivated field_1 and field_2 soils were closely related to humus and water content, respectively. Further RDA identified the families and major SVs that were strongly associated with each chemical parameter (Supplementary Fig. S4a–d and Table S4). The Mucoraceae family and two families in Actinomycetota were associated with humus; the Euglyphida family with pH; and three families in Ascomycota with nitrate nitrogen. Notably, many Cercozoa- and Ascomycota-derived SVs were associated with soil pH and nitrite nitrogen, nutrient ions, respectively. Associations between prokaryotic SVs in particular phyla or families and chemical factors were observed: Actinomycetota- and Pseudomonadota-derived SVs with nitrate nitrogen; the Pyrinomonadaceae, Gemmatimonadaceae, Chthoniobacteraceae, Pedosphaeraceae, WD2101_soil_group and SC-I-84 families with nutrient ions; and maize (SV_1) and cabbage (SV_3) with nitrite nitrogen and soil pH, respectively.

### Eukaryotic and prokaryotic networks in field soils under crop rotation

#### Network structures and phyla of the node SVs

**Figure 5:**
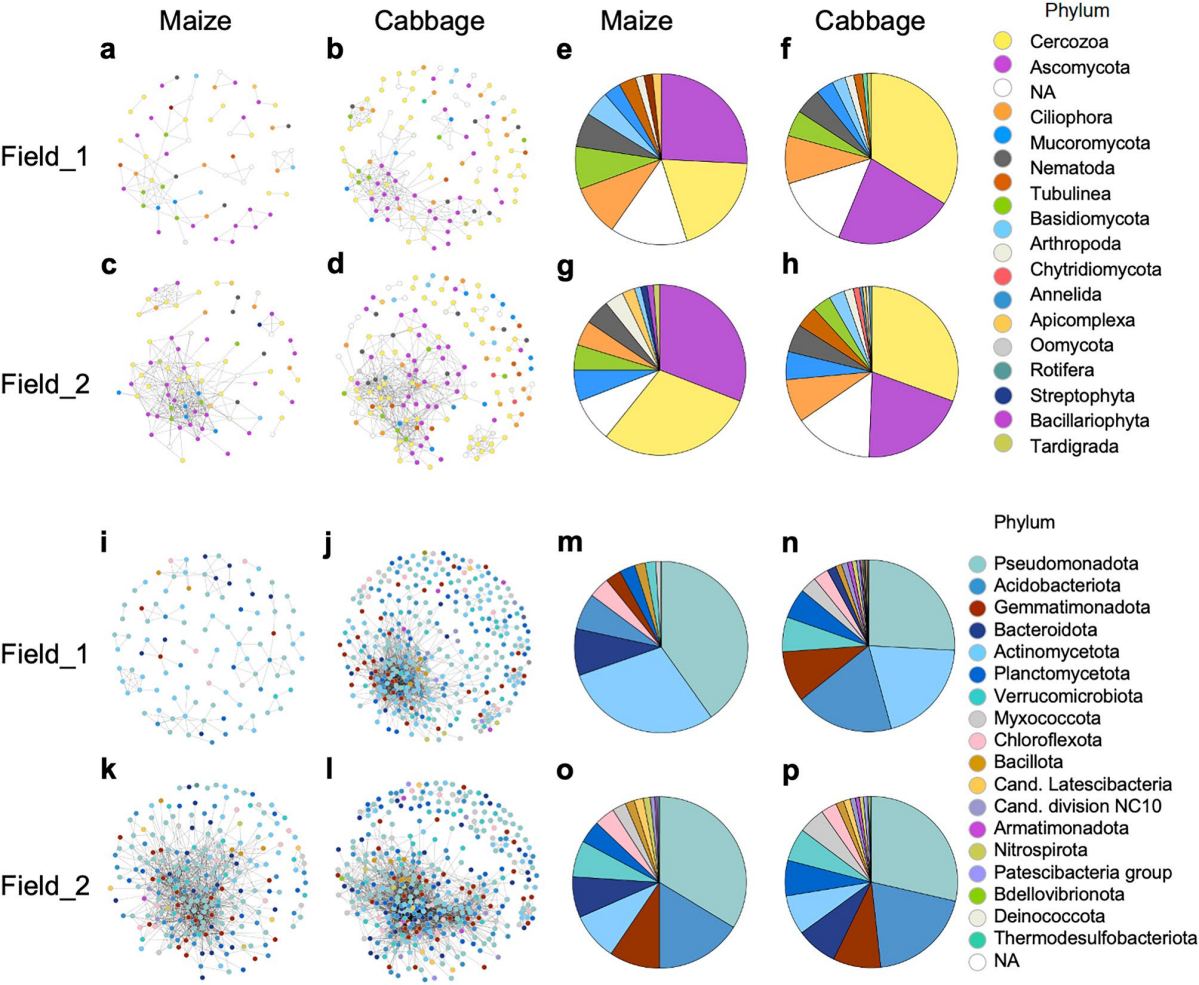
Networks of eukaryotic and prokaryotic SVs and the corresponding pie charts of the relative abundance of the phyla of the SVs in each sample group. Networks of eukaryotic and prokaryotic SVs are derived from field_1 cropping maize (**a**,**i**) and cabbage (**b**,**j**), and field_2 cultivating maize (**c**,**k**) and cabbage (**d**,**l**), respectively. The enlarged diagrams containing SVs are shown in Supplementary Fig. S5a–h. Pie charts of the corresponding eukaryotic and prokaryotic networks represent the results from field_1 cropping maize (**e**,**m**) and cabbage (**f**,**n**), and field_2 cultivating maize (**g**,**o**) and cabbage (**h**,**p**), respectively. Eukaryotic and prokaryotic phyla of nodes (nSVs) and pie charts are indicated by colors as shown in the upper and lower right, respectively. *NA* not assigned phylum.

To investigate the biological interactions among soil organisms, we analyzed the networks of eukaryotic and prokaryotic SVs based on the *ϕ* coefficient values in the four sample groups (Supplementary Table S5). The eight network diagrams of node SVs (nSVs) were prepared by the data from the maize- and cabbage-cultivated field_1 and field_2 soils (Fig. 5a–d for eukaryotic nSVs and i–l for prokaryotic nSVs; Supplementary Fig. S5a–h). These diagrams indicate that the complexity of networks was apparently increased in the cabbage-cultivated field soils, especially in field_1 soils (Fig. 5a,b vs. Fig. 5i,j). In addition, the eukaryotic and prokaryotic networks in the maize-cultivated field_1 soils were poorly formed by comparison with those in the other three sample groups in terms of the number of links, nodes (nSVs), and maximum cluster sizes (Fig. 5a,i, Supplementary Table S6). The relative abundances of nSVs at the phylum level in the four sample groups are shown by pie charts (Fig. 5e–h and m–p) and Supplementary Table S7. Many nSVs were derived from two eukaryotic phyla Ascomycota and Cercozoa, and four prokaryotic phyla Pseudomonadota, Acidobacteriota, Actinomycetota and Gemmatimonadota. Interestingly, the most abundant eukaryotic phyla changed from Ascomycota to Cercozoa after the rotation from maize to cabbage in two field soils (Fig. 5e,g vs. Fig. 5f,h). The relative abundance of Ascomycota-, Basidiomycota-, and Oomycota-derived nSVs all decreased with the maize-to-cabbage rotation (Supplementary Table S7). In prokaryotes, the Actinomycetota-derived nSVs were markedly dominant in field_1 soils compared with those in field_2 soils. The relative abundance of nSVs from Acidobacteriota, Gemmatimonadota, Planctomycetota, Verrucomicrobiota, and Myxococcota phyla were lower in the maize-cultivated field_1 soils than those in other soils, and nSVs of three phyla (Acidobacteriota, Planctomycetota, and Myxococcota) were all increased in the cabbage-cultivated field soils.

We then investigated the clusters of nSVs in the network and their nSVs phyla in four sample groups (Supplementary Figs. S6 and S7). In eukaryotic networks, a single large cluster containing 33–97 nSVs were detected together with 10–28 minor clusters in each network (Supplementary Fig. S6), which mainly contained the Ascomycota- and Cercozoa-derived nSVs and phylum-unassigned nSVs. Similar clusters (i.e., a single large cluster of 203–292 nSVs plus 10–25 small clusters) were found in prokaryotic networks and contained the Pseudomonadota-, Acidobacteriota-, Actinomycetota- and Gemmatimonadota-derived nSVs, except for those in the maize-cultivated field_1 soils that lacked any large clusters (Supplementary Fig. S7a).

#### Core node SVs in the four sample groups

We then identified the top 10% of eukaryotic and 5% of prokaryotic nSVs using link numbers as “core nSVs” because of their potentially crucial roles in network formation. The link numbers and phyla of these nSVs (Supplementary Table S8), their numbers and percentage in total nSVs (Supplementary Table S9) were summarized for each sample group. SV_4 was shared by only four sample groups among eukaryotic core nSVs and three (SV_2, 28, and 84) and two (SV_43 and 63) eukaryotic core nSVs were commonly found in the maize- and cabbage-cultivated soils, respectively. In addition to SV_4, SV_2 and SV_28 were found in field_2 soils. Conversely, any prokaryotic core nSVs were not shared by either the crop-cultivated soils or the field soils except for SV_23 and SV_88 found in field_2 soils (Supplementary Table S8). The relative abundance of two fungi (Basidiomycota and Mucoromycota)-derived core nSVs decreased while that of the protist (Cercozoa and Ciliophora)-derived core nSVs increased after rotation to cabbage cultivation (Supplementary Table S9). In prokaryotes, the Actinomycetota-derived core nSVs were exclusively detected in field_1 soils. The relative abundance of Pseudomonadota-derived core nSVs decreased and those of core nSVs from the phyla Gemmatimonadota, Candidatus Latescibacteria, and Planctomycetota increased under cabbage cultivation.

Finally, we analyzed the nSVs commonly shared by 4 sample groups (i.e., common nSVs) because of their potential role in the network core and identified 13 eukaryotic and 7 prokaryotic common nSVs with their taxa (Supplementary Tables S10 and S11). Nine of thirteen eukaryotic common nSVs were derived from fungi (Supplementary Table S10), and five of seven prokaryotic common nSVs belonged to the Pseudomonadota phylum (Supplementary Table S11). These common nSVs mostly belonged to the largest cluster (cluster 1) in the corresponding sample groups, except for the maize-cultivated filed_1 samples. Many eukaryotic common nSVs, especially the protist- and two Basidiomycota-derived common nSVs, were also core nSVs, but this was not the case for prokaryotic common nSVs. In addition, we detected 4 and 8 maize-cultivated and 18 and 52 cabbage-cultivated soil-specific eukaryotic and prokaryotic nSVs, respectively (Supplementary Tables S10 and S11), of which the former nSVs were present in the networks of the maize-cultivated soils and disappeared in those of the cabbage-cultivated soils, and vice versa for the latter nSVs. Notably, 10 of the 16 phylum-known cabbage-cultivated soil-specific nSVs were derived from protist phyla (Cercozoa, Tubulinea, and Ciliophora). In prokaryotic nSVs, the Actinomycetota-derived nSVs were abundantly found as field_1 soil-specific nSVs. In addition, five and seven genera-derived prokaryotic nSV groups with more than three members were found in the cabbage-cultivated soil-specific nSVs (*Chloracidobacterium*, *Luteitalea*, *Paludibaculum* in Acidobacteriota, *Rhabdothermincola* in Actinomycetota, and *Humisphaera* in Planctomycetota) and in field_2 soil-specific nSVs (*Luteitalea* and *Pyrinomonas* in Acidobacteriota, *Tepidiforma* in Chloroflexota, *Humisphaera* in Planctomycetota, *Sphingomonas* in Pseudomonadota, and *Chthoniobacter* and *Pedosphaera* in Verrucomicrobiota) (Supplementary Table S11).

## Discussion

Several DNA metabarcoding studies have analyzed soil bacterial and fungal communities in agricultural lands under various types of crop rotation, but understanding remains poor concerning other eukaryotes. In addition, few studies have assessed the soil biota under poaceous crops-brassicaceous crop rotation^7,14,23,24,31^. Therefore, we investigated both eukaryotes and prokaryotes and their networks formed in two agricultural fields in central Japan under the 1st and 2nd cycles of maize–cabbage rotation.

The major phyla of SVs found in our study were Ascomycota, Basidiomycota, and Cercozoa in eukaryotes and Pseudomonadota, Acidobacteriota, and Actinomycetota in prokaryotes (Fig. 2). These fungal and bacterial phyla have been commonly detected in other studies on crop rotations^9,12,18,22,24,25,27,32,33,36,37^. We found different bacterial phylum compositions between two field soils, where the relative abundance of the Actinomycetota phylum in field_1 soils was clearly higher than that in field_2 soils (Fig. 2b). The abundant Actinomycetota-derived nSVs were consistently found in the prokaryotic networks (Fig. 5m,n). Previous studies showed that the abundance of bacteria in Actinomycetota is increased by treatment with green manure^38–40^, and Tao et al. also reported that green manure fertilization altered the topological properties of microbial networks^41^. These studies are consistent with our observations because of the green manure supplied to field_1 in the previous year. Several families were unequally distributed according to field and crop, and their relative abundances were changed by cabbage cultivation (Supplementary Table S1). Fungal Mrakiaceae and protist Euglyphida families were more abundant in field_2 vs. field_1 and their relative abundances were differently changed by crop rotation to cabbage. The biased relative abundance of some families was likely accounted for by the different contents of chemical factors in the fields; for example, the Mrakiaceae (SV_2) and Sphingomonadaceae families, which were more abundant in field_2 vs. the other field, were strongly associated with water content in RDA, which was high in field_2 (Fig. 4a, Supplementary Table S4). The prokaryotic families and genera for which the relative abundances were increased by crop rotation in both fields, such as family Vicinamibacteraceae and field_2 families Nitrosomonadaceae and Pyrinomonadaceae and genus *RB41*, contained cabbage- and field_2-specific nSVs, respectively. The Chitinophagaceae and Rhodanobacteraceae families and genus *Nocardioides*, the abundances of which were decreased in both fields, tended to have maize-specific nSVs. These results suggest the involvement of those nSVs in the changes of networks triggered by cabbage cultivation.

The maize–cabbage rotation in the fields is used to suppress serious clubroot diseases of cabbage^35^. We identified the SV_97 derived from *P. brassicae*, which is a pathogenic protist causing clubroot diseases^42^. SV_97 was abundantly detected in the maize-cultivated field_2 soils under the 2nd rotation cycle in 2019, where cabbage was planted in 2018. The relative abundance of SV_97 gradually decreased during maize growth and was significantly reduced in the cabbage-cultivated field_2 soils. These results indicate that *P. brassicae* that propagated during cabbage cultivation in 2018 was detected in the maize-cultivated soils in 2019, and further expansion of the pathogen was likely suppressed by the practices applied during the maize harvest, i.e., treatments with anti-clubroot disease agent flusulfamide, chemical fertilizer supply, and tillage with maize residues. In addition to effective flusulfamide treatment, precropped maize may contribute to reduce the density of pathogens in the cabbage-cultivated field_2 soils by trapping *P. brassicae* with roots because maize does not exhibit a clubroot phenotype. Changes in soil microbial diversity by crop rotation (Fig. 3b) may also influence the suppression of clubroot disease via modulation of the pathogen transcriptomes, as reported by Daval et al.^43^.

Regarding the communities in soils under crop rotation, many studies have indicated distinct β-diversities of soil bacteria and/or fungi between mono- and rotation-cropping^5,6,8,10,12,14–18,20–22,24,25,27,33^ or among crop rotations with different crops and rotation sequences, places, and/or periods^5,10,11,15,18–22,24,25,32,33^; however, the details of soil biota that change with plant growth have not been clarified in a crop-rotation cycle. We showed that the taxonomic variations indicated by β-diversities in the four sample groups were clearly distinguished by crops as well as by fields (Fig. 3), suggesting the presence of unique eukaryotic and prokaryotic communities in each soil. This result is unsurprising because microbial communities including fungi are known to be influenced by crops and land use history, including crop rotations. This is also the case with eukaryotic communities; for instance, protist communities were changed by fertilization^44^ and depth^45^, and unique communities of eukaryotes^46–49^, including protists^50–53^ and nematodes^54,55^, were formed in different types of soils, including agricultural soils. Furthermore, the taxonomic variations in β-diversities were almost comparable among the three different growth stages of crops except for prokaryotic variations in field_2 soils cropping maize at the early stage (Fig. 3), suggesting that the communities in bulk soils near crops are largely unaffected by plant growth. We also found higher Shannon indexes of eukaryotes for α-diversity in the cabbage-cultivated field soils vs. maize-cultivated soils (Supplementary Fig. S3a), which may reflect the appearance or propagation of additional species in eukaryotic communities via the cultivation of crops of different families. In particular, the low Shannon index in field_1 soils significantly increased to levels comparable with those in field_2 soils after crop rotation to cabbage (Supplementary Table S2). This observation may be accounted for by a study reporting a higher OTU richness of fungi and protists in agricultural fields compared with grasslands and woods^47^: because of fallow in field_1 in the previous year, the Shannon index in the maize-cultivated field_1 soils was low, and then increased with subsequent cabbage cultivation. Conversely, the prokaryotic α-diversities in field_1 soils cropping maize were high and decreased to the levels of those in field_2 soils with subsequent cabbage cultivation (Supplementary Fig. S3b). This could also be explained by the previous observation reported by Woo and colleagues, who showed that the Shannon index of bacteria in fallow was highest compared with those observed in mono- and rotationally cropped fields with pea and wheat^28^. These data suggest that the distinct communities in the two field soils were formed by different agricultural practices (i.e., fallow with green manure and rotation cropping) before maize cultivation and that a rapid change in eukaryotic and prokaryotic communities in field_1 soils was caused by the first cropping.

RDA revealed that the eukaryotes and prokaryotes in field_2 soils were closely related to nutrient ions and phosphorus content, and those in the maize-cultivated field_2 soils to water content (Fig. 4). Despite minor differences among investigations, previous studies on crop rotations, including maize or cabbage, showed that soil pH and nutrients (mainly nitrogen) often affect bacterial and/or fungal communities^12,17,18,20,23,25,33,36,56^. The higher content of these nutrients and of water in field_2 compared with those in field_1 may account for the above relationships (Fig. 4a). In field_1, the communities in the maize-cultivated soils were related to the humus content, which was likely derived from green manure supplied in the previous year. Regarding the relationship to crops, the communities in the maize-cultivated soils were associated with ammonium and nitrite nitrogen content, and electrical conductivity, and those in the cabbage-cultivated soils to soil pH. Similar RDA profiles of both prokaryotes and eukaryotes were obtained, indicating that the eukaryotic communities are affected by soil chemical factors in a similar manner to prokaryotic communities. RDA identified particular families and major SV groups that were strongly associated with each chemical parameter (Supplementary Table S4). For instance, cabbage- and Cercozoa-derived SVs and maize-, Ascomycota- and Nematode-derived SVs were associated with pH and nitrate nitrogen, respectively, indicating that each chemical factor affected each crop and SV group. Moreover, many cabbage-specific nSVs were found in nonplant SVs associated with pH (5 of 11), suggesting that soil pH contributes to the formation of complex eukaryotic networks in cabbage-cultivated soils as described later. Similarly, many eukaryotic and prokaryotic nSVs among the SVs associated with nutrient ions were field_2-specific (5 out of 11 and 11 out of 25 SVs, respectively), and four field_2-specific nSVs out of six prokaryotic SVs were associated with water content. Because of the high content of nutrient ions and water in field_2, these factors may have influenced the nSVs during network formation in field_2.

Finally, we investigated the prokaryotic and eukaryotic networks in each sample group. Notably, networks where poorly formed with small numbers of nSVs and links in the maize-cultivated field_1 soils where the first rotation cycle was initiated compared with those in field_2 soils under the second rotation cycle (Fig. 5, Supplementary Table S6), suggesting distinct communities in two field soils under maize cultivation as mentioned above. Under cabbage cultivation, complex networks were formed in field_1 soils as well as in field_2 soils. Furthermore, despite a few core nSVs being shared by the networks in the crop-cultivated or the field soils, most of the eukaryotic and prokaryotic core nSVs were not conserved in the networks throughout the maize–cabbage crop rotation in each field (Supplementary Table S8). These observations indicate distinct networks in the maize- and cabbage-cultivated soils in each field and are consistent with the β-diversity results (Fig. 3), suggesting that the communities and networks in the field soils were easily changed and affected by cropping. Several studies have shown that rotation cropping produces more complex networks (mainly bacteria and fungi) than monoculture does^12,13,18^ and that the microbial networks are distinct among different crop rotations^19^. Crops and agricultural management practices such as fertilization and green manures also affect microbial communities and their networks^41,57–62^. Xiong et al. showed a change in microbial networks along with maize developmental stages^63^. Xie et al. reported different microbial networks in each crop under wheat–rice rotation as we observed^64^. These studies suggest that the networks of soil organisms are flexibly reformed by coupling with the changes in soil biota upon crop cultivation and/or soil environmental changes and agree with our observations. Our data suggest the increase in the complexity of networks by cropping with cabbage. Based on previous studies, subsequent cropping with plants in different families (maize in *Poaceae* and cabbage in *Brassicaceae*) and/or tillage with maize residues may contribute to the increased complexity of networks triggered by cabbage cultivation via newly generated plant–soil organism interactions.

We identified 13 eukaryotic and 7 prokaryotic common nSVs shared by the networks of 4 sample groups (Supplementary Tables S10 and S11), and these Ascomycota- or Cercozoa-derived and Pseudomonadota-derived common nSVs likely have potential roles in forming core networks. We also identified core nSVs for nSVs because of the crucial role of hubs in the network. Few crop- or field-specific eukaryotic core nSVs were present in the nSVs; however, five eukaryotic SVs (SV_2, 4, 28, 43, and 63) were not only common nSVs but also core nSVs in more than two networks (Supplementary Table S10). Two abundant SVs (SV_2 and SV_4) were derived from the Basidiomycota *Tausonia* and *Solicoccozyma* genera that respectively contains enzyme-producing yeasts^65^ and plant-growth promoting microorganisms^66^. SV_28, SV_43, and SV_63 were assigned to the *Spongomonas* and *Heteromita* genera in Cercozoa and the *Colpoda* genus in Ciliophora respectively. *Heteromita* is an abundant flagellate^67^, *Spongomonas* and *Heteromita* are bacterivorous protists^68^, *Colpoda* is a common free-living terrestrial protist, and *Colpoda cucullus* has been reported to improve maize growth^69^. These common SVs could act as core hubs of eukaryotic networks in both fields throughout cropping. By contrast, only one of the seven prokaryotic common nSVs (SV_23) was a core nSV in the two networks. Furthermore, although six core nSVs (SV_2, 4, 28, 43, 63, and 84) were conserved in the eukaryotic networks of the maize- or cabbage-cultivated soils, no conserved core nSVs were found in the corresponding prokaryotic networks (Supplementary Table S8). This may suggest that networks of soil prokaryotes are more unstable than those of eukaryotes under crop rotation.

Protists are an important group of soil eukaryotes^1^ and are involved in nutrient cycles in the pedosphere and in plant growth and health in agricultural lands^70^; however, protists have not been well characterized compared with soil bacteria or fungi. Notably, the numbers of core nSVs, especially protist (phyla Cercozoa and Ciliophora)-derived core nSVs in this study, were markedly increased in the eukaryotic networks under cropping cabbage (Supplementary Table S9). Several studies have been conducted on nonfungal eukaryotic networks, including those of protists^52,53,59,71^ and nematodes^72^, in agricultural soils, and some of these demonstrated the ecological importance of bacteria-fungi-protist networks in the rhizosphere^52,59^, of fungi-protist interactions in paddy soils^71^, and of protist networks in arable soils^53^. Our observations also showed that protists helped increase the complexity of eukaryotic networks in the cabbage-cultivated soils. Protists are drivers for modifying soil microbiomes, and it is therefore important to clarify the taxonomic compositions of whole soil organisms and dynamic changes in their networks for advanced crop cultivation in the future.

In conclusion, we have showed that both chemical parameters and crops affect eukaryotic and prokaryotic communities and clarified the structures and changes in eukaryotic and prokaryotic networks under maize–cabbage crop rotation. We have newly demonstrated the involvement of protists in eukaryotic network formation.

## Methods

### Study sites and soil sampling

This study was performed in two closely-located agricultural fields (i.e., field_1 and field_2), in Tahara city (Aichi prefecture) in central Japan (34.61° N, 137.07° E) (Fig. 1a). Field_1 (2000 m^2^ in area and 3 m in altitude) and field_2 (500 m^2^ and 6 m) were a paddy and a red soil-rich field, respectively, and both field soils were replaced with sandy-gravelly soils for soil improvement. Both fields had comparable properties of soils and agricultural practice (supply of water, organic and chemical fertilizers, and anti-clubroot agents and tillage). Since 2003, monocropping with cabbage (*Brassica oleracea*) or rotation cropping with maize (*Zea mays*) in spring and cabbage in fall have been performed in the two fields. In both fields, maize and cabbage were cultivated by rotation during May to June and September to November in 2019, respectively. The maze–cabbage rotation was performed at field_2 with two second rotation cycles (2018 and 2019) and at field_1 in 2019 while the first rotation cycle in field_1 was left fallow and tilled with Sorghum as green manure in 2018 (Fig. 1b). The fields were tilled and furrowed (70 cm-distance between ridges) before cropping. Crops were cultivated (35 cm-distance between plants) and grown on a ridge covered with a white mulching sheet. Fertilizer and water were applied equally, and the growth of crops was comparable between the two fields. In the crop rotation, chemical fertilizer was supplied to tilled soils before maize cultivation. After the maize harvest, the field soils were mixed with maize residues by tillage, supplied with chemical fertilizers, and treated with the fungicide flusulfamide (Nebijin, Mitsui Chemicals Agro, Tokyo) for suppressing clubroot disease of cabbage^35^. Surface soils near the plants (within 10 cm-distance from the plants and 15 cm in depth) were sampled at three different stages of plant growth (i.e., early, middle, and late stages) under clear climatic conditions on May 20, June 4, and 26 in field_1, and May 7, May 20, and June 17 in field_2 for maize (note: soil sampling was performed at comparable growth stages of maize in each field because of the slightly different planting time), and September 12, October 7, and November 5 for cabbage in 2019. More than four soil samples were isolated from near the plant and mixed together at each sampling point, where the neighbored sampling point was located 35 cm apart. A total of 36 sample soils were isolated from 3 independent sampling points at 2 fields cultivated with 2 crops at 3 growth stages and pretreated before DNA purification, as described previously^34^. This study was carried out in accordance with relevant guidelines.

### Soil DNA purification and rRNA amplicon sequencing

Whole soil DNA was purified from 10 g of fresh soil using the DNeasy PowerMAX Soil Kit (QIAGEN, Venlo, Netherland), and 400-μL aliquots were concentrated to 50 μL in TE buffer (pH 8.0) by ethanol precipitation with 40 μL of 3 M sodium acetate (pH 5.2) and 1 mL of ethanol. Purified soil DNA was stored at − 20 °C. The 16S (V3–V4 region) and 18S (V7–V8 region) rRNA genes were amplified from soil DNA with the universal primers 16S_Amplicon_MiseqF and 16S_Amplicon_MiseqR (341F and 805R with tail sequences^73^) and F1183-18S_V7-V8_MiseqF and R1631a-18S_V7-V8_MiseqR^34^, respectively. The PCR mixture (20 μL) contained 10 μL of 2 × Buffer for KOD FX Neo, 4 μL of 2 mM dNTPs, 0.4 units of KOD FX Neo DNA polymerase (Toyobo, Tokyo, Japan), 2 μL of template DNA, and 0.3 mM each of the forward and reverse primers. Amplification was initiated with denaturation at 94 °C for 2 min followed by 30 cycles of denaturation at 94 °C for 10 s, annealing at 55 °C for 30 s, and extension at 68 °C for 60 s. Amplified PCR products were purified with 0.8 volume of AMPure XP beads (Beckman Coulter, Brea, California, USA) and eluted with 10 mM Tris–HCl (pH 8.5). Index PCR was performed in eight cycles using a Nextera XT Index Kit v2 (Illumina, San Diego, California, USA). The amplified libraries were purified by the addition of 1.12 volumes of AMPure XP beads and eluted with 10 mM Tris–HCl (pH 8.5). Library concentrations were quantified using a spectrophotometer, and equal amounts of each library were pooled and quantified using a Qubit dsDNA HS Assay Kit (Thermo Fisher Scientific, Waltham, Massachusetts, USA). Each 250-bp end of the pooled library was sequenced using an MiSeq Reagent Kit v2 (500 cycles; Illumina) on the MiSeq instrument (Illumina). Sequences were deposited in the DDBJ Sequence Read Archive (DRA) database under the accession numbers DRR428059-DRR428130.

### Sequence data analysis and bioinformatics

The sequence data of 16S and 18S rRNA genes from 36 amplicons were independently imported into QIIME2 version 2021.4^74^ and the primer sequences were removed using the Cutadapt plugin (version 3.4)^75^ with the default parameters. Forward and reverse reads were joined, denoised, and chimera checked using the dada2 plugin^76^. The resultant SVs of 16S and 18S rRNA genes (i.e., prokaryotic and eukaryotic SVs, respectively) were further processed by vsearch (version 2.7.0)^77^ uchime ref command with a minimum score option of—minh 0.5 for removal of chimeric sequences. The taxonomic assignment of the SVs was based on the SILVA database (version 138)^78^, with a 99% clustering threshold (note: some taxa such as the order Euglyphida were incorrectly classified in the SILVA database). Some SILVA-derived taxa were corrected based on the taxonomy data in the National Center for Biotechnology Information (https://www.ncbi.nlm.nih.gov/taxonomy). Finally, the 16S and 18S rRNA gene-derived SVs < 400 and < 407-bp in length were removed, respectively, and the remaining SVs were used for further analyses. The phylum-, family- and genus-level compositions of the major eukaryotic and prokaryotic SVs (> 0.5% and > 1% of the total reads, respectively) were displayed as histograms for each sample using the R packages phyloseq (v1.32.0)^79^ and ggplot2 (v3.3.3). For α- and β-diversity analyses, SVs were rarefied using the R phyloseq package’s rarefy_even_depth function and the Shannon index plot for α-diversity was obtained using plot_richness functions in the phyloseq package in R. Difference of the Shannon indexes between two samples was investigated by Tukey–Kramer test in R. The NMDS plot of the Bray–Curtis distance matrix for β-diversity was obtained using ordinate and plot_ordination functions in the R package phyloseq. The adonis2 (Permanova) test^80^ based on Bray–Curtis distance matrices was performed to determine whether there were significant differences in sample groups (https://search.r-project.org/CRAN/refmans/vegan/html/adonis.html). For RDA and network analysis, the logarithm ratio data was obtained with cardinal log-ratio transformation (*clr*) using geometric means of read numbers of total SVs in the sample group and the aldex.clr function in the ALDEx2 package (https://www.rdocumentation.org/packages/ALDEx2/versions/1.4.0/topics/aldex.clr). RDA was performed using the rda function in the R package vegan to investigate the relationship between chemical properties and prokaryotic and eukaryotic taxa in each sample, and families and major SVs (top 350 in abundance). The network analysis was performed based on the *ϕ* coefficient between SVs by applying the propr.aldex.phi function in R created by Greg Gloor to the log-ratios (https://github.com/ggloor/CoDa_microbiome_tutorial/blob/master/chunk/R/propr-functions.R), and the relationships of eukaryotic and prokaryotic SVs with the *ϕ* coefficient of < 0.12 and < 0.08 were investigated, respectively. The resultant network diagrams and pie charts of relative abundance of nSVs phyla were visualized with the igraph package in R. Sequence identity analysis of several nSVs were performed using the blastn program against the GenBank database in August 2022 via the internet (https://blast.ncbi.nlm.nih.gov/Blast.cgi), and the taxonomic information of the genus-identified hits with the lowest e-value was used as taxa of those SVs. For heatmap visualization of the chemical data in the samples, the means of measured values from the triplicates were determined in 12 samples from different growth stages, crops, and fields, and the percentage of each sample was calculated by the mean value divided by the total values of the mean values of 3 samples in each chemical parameter; a heatmap was prepared by clustering with the farthest neighbors algorism using the heatmap function in R.

### Soil chemical parameters

The following parameters were measured using a triplicate sample soils dried at 60 °C overnight by Inochio Agricultural Central Research Center (Aichi, Japan): cation-exchange capacity, pH in water, nitrate nitrogen, ammonium nitrogen, exchangeable potassium, exchangeable magnesium, exchangeable calcium, electric conductivity, available phosphorus, and humus contents. Water content was determined as the ratio of water in soil: the weight subtracted from fresh soil by dried soil was divided by the weight of fresh soil.

### Supplementary Information

Supplementary Information.

Supplementary Table S5.

## Data Availability

Sequence data were deposited in the DDBJ Sequence Read Archive (DRA) database under the accession numbers DRR428059-DRR428130. The information on the relative abundance of SVs at the family and genus levels, statistical analysis of α-diversity, the families and SVs associated with the chemical parameters, soil chemical parameter data, phi value data in the network analysis, and the details of node SVs and core node SVs in the four sample groups are provided in the Supplementary Tables.

## References

[1] Potapov AM (2022). Feeding habits and multifunctional classification of soil-associated consumers from protists to vertebrates. Biol. Rev. Camb. Philos. Soc..

[2] Toju H (2018). Core microbiomes for sustainable agroecosystems. Nat. Plants.

[3] Larkin RP (2015). Soil health paradigms and implications for disease management. Annu. Rev. Phytopathol..

[4] Bahram M (2018). Structure and function of the global topsoil microbiome. Nature.

[5] Ai C (2018). Distinct responses of soil bacterial and fungal communities to changes in fertilization regime and crop rotation. Geoderma.

[6] Behnke GD (2021). Soil microbial indicators within rotations and tillage systems. Microorganisms.

[7] Blakney AJC, Bainard LD, St-Arnaud M, Hijri M (2022). Brassicaceae host plants mask the feedback from the previous year's soil history on bacterial communities, except when they experience drought. Environ. Microbiol..

[8] Breidenbach B, Brenzinger K, Brandt FB, Blaser MB, Conrad R (2017). The effect of crop rotation between wetland rice and upland maize on the microbial communities associated with roots. Plant Soil.

[9] Cezar RM (2021). Crop rotation reduces the frequency of anaerobic soil bacteria in Red Latosol of Brazil. Braz. J. Microbiol..

[10] Fadiji AE, Kanu JO, Babalola OO (2021). Metagenomic profiling of rhizosphere microbial community structure and diversity associated with maize plant as affected by cropping systems. Int. Microbiol..

[11] Hoeffner K (2021). Legacy effects of temporary grassland in annual crop rotation on soil ecosystem services. Sci. Total Environ..

[12] Jiang Y (2022). Rotation cropping and organic fertilizer jointly promote soil health and crop production. J. Environ. Manag..

[13] Jiang YJ (2016). Crop rotations alter bacterial and fungal diversity in paddy soils across East Asia. Soil Biol Biochem..

[14] Kerdraon L, Balesdent MH, Barret M, Laval V, Suffert F (2019). Crop residues in wheat-oilseed rape rotation system: A pivotal, shifting platform for microbial meetings. Microb. Ecol..

[15] Kracmarova M (2022). Soil microbial communities following 20 years of fertilization and crop rotation practices in the Czech Republic. Environ. Microbiome.

[16] Li H (2022). Impacts of continuous and rotational cropping practices on soil chemical properties and microbial communities during peanut cultivation. Sci. Rep..

[17] Liu JJ (2017). Distinct soil bacterial communities in response to the cropping system in a Mollisol of northeast China. Appl. Soil Ecol..

[18] Liu ZX (2020). Long-term continuous cropping of soybean is comparable to crop rotation in mediating microbial abundance, diversity and community composition. Soil Till. Res..

[19] Lu J (2022). The impact of different rotation regime on the soil bacterial and fungal communities in an intensively managed agricultural region. Arch. Microbiol..

[20] Lyu J (2020). Effects of different vegetable rotations on fungal community structure in continuous tomato cropping matrix in greenhouse. Front. Microbiol..

[21] Maarastawi SA, Frindte K, Linnartz M, Knief C (2018). Crop rotation and straw application impact microbial communities in Italian and Philippine soils and the rhizosphere of *Zea*
*mays*. Front. Microbiol..

[22] Niu J (2017). Insight into the effects of different cropping systems on soil bacterial community and tobacco bacterial wilt rate. J. Basic Microbiol..

[23] Qiao CC (2019). Reshaping the rhizosphere microbiome by bio-organic amendment to enhance crop yield in a maize–cabbage rotation system. Appl. Soil Ecol..

[24] Shen J (2022). The exacerbation of soil acidification correlates with structural and functional succession of the soil microbiome upon agricultural intensification. Sci. Total Environ..

[25] Soman C, Li DF, Wander MM, Kent AD (2017). Long-term fertilizer and crop-rotation treatments differentially affect soil bacterial community structure. Plant Soil.

[26] Srour AY (2020). Microbial communities associated with long-term tillage and fertility treatments in a corn-soybean cropping system. Front. Microbiol..

[27] Su Y (2022). Contrasting assembly mechanisms and drivers of soil rare and abundant bacterial communities in 22-year continuous and non-continuous cropping systems. Sci. Rep..

[28] Woo SL (2022). Pea–wheat rotation affects soil microbiota diversity, community structure, and soilborne pathogens. Microorganisms.

[29] Wu X (2021). Deciphering microbial mechanisms underlying soil organic carbon storage in a wheat-maize rotation system. Sci. Total Environ..

[30] Xi H (2021). Effects of cotton-maize rotation on soil microbiome structure. Mol. Plant Pathol..

[31] Yang XX (2020). Effects of different rotation patterns on the occurrence of clubroot disease and diversity of rhizosphere microbes. J. Integr. Agric..

[32] Yu H (2021). Effects of rotations with legume on soil functional microbial communities involved in phosphorus transformation. Front. Microbiol..

[33] Zhang Y, Li W, Lu P, Xu T, Pan K (2022). Three preceding crops increased the yield of and inhibited clubroot disease in continuously monocropped Chinese cabbage by regulating the soil properties and rhizosphere microbial community. Microorganisms.

[34] Kenmotsu H, Takabayashi E, Takase A, Hirose Y, Eki T (2021). Use of universal primers for the 18S ribosomal RNA gene and whole soil DNAs to reveal the taxonomic structures of soil nematodes by high-throughput amplicon sequencing. PLoS One.

[35] Dixon GR (2009). The occurrence and economic impact of *Plasmodiophora brassicae* and clubroot disease. J. Plant Growth Regul..

[36] Alami MM (2021). Continuous cropping changes the composition and diversity of bacterial communities: A meta-analysis in nine different fields with different plant cultivation. Agriculture (Basel).

[37] Navarro-Noya YE (2022). Bacterial communities in the rhizosphere at different growth stages of maize cultivated in soil under conventional and conservation agricultural practices. Microbiol. Spectr..

[38] Gao SJ, Cao WD, Zhou GP, Rees RM (2021). Bacterial communities in paddy soils changed by milk vetch as green manure: A study conducted across six provinces in South China. Pedosphere.

[39] Ozbolat O (2023). Long-term adoption of reduced tillage and green manure improves soil physicochemical properties and increases the abundance of beneficial bacteria in a Mediterranean rainfed almond orchard. Geoderma.

[40] Yang R, Song SJ, Chen SY, Du ZY, Kong JQ (2023). Adaptive evaluation of green manure rotation for a low fertility farmland system: Impacts on crop yield, soil nutrients, and soil microbial community. CATENA.

[41] Tao J (2018). Integrated network analysis reveals the importance of microbial interactions for maize growth. Appl. Microbiol. Biotechnol..

[42] Schwelm A (2018). Not in your usual Top 10: Protists that infect plants and algae. Mol. Plant Pathol..

[43] Daval S (2020). Soil microbiota influences clubroot disease by modulating *Plasmodiophora brassicae* and *Brassica napus* transcriptomes. Microb. Biotechnol..

[44] Xiong W (2018). Soil protist communities form a dynamic hub in the soil microbiome. ISME J..

[45] Roy J, Mazel F, Dumack K, Bonkowski M, Rillig MC (2022). Hierarchical phylogenetic community assembly of soil protists in a temperate agricultural field. Environ. Microbiol..

[46] Abujabhah IS, Bound SA, Doyle R, Bowman JP (2016). Effects of biochar and compost amendments on soil physico-chemical properties and the total community within a temperate agricultural soil. Appl. Soil Ecol..

[47] George PBL (2019). Divergent national-scale trends of microbial and animal biodiversity revealed across diverse temperate soil ecosystems. Nat. Commun..

[48] Matus-Acuna V, Caballero-Flores G, Martinez-Romero E (2021). The influence of maize genotype on the rhizosphere eukaryotic community. FEMS Microbiol. Ecol..

[49] Lammel DR, Nusslein K, Cerri CEP, Veresoglou SD, Rillig MC (2021). Soil biota shift with land use change from pristine rainforest and Savannah (Cerrado) to agriculture in southern Amazonia. Mol. Ecol..

[50] Taerum SJ (2022). 18S rRNA gene amplicon sequencing combined with culture-based surveys of maize rhizosphere protists reveal dominant, plant-enriched and culturable community members. Environ. Microbiol. Rep..

[51] Schulz G (2019). Changes in trophic groups of protists with conversion of rainforest into rubber and oil palm plantations. Front. Microbiol..

[52] Hilton S (2021). Identification of microbial signatures linked to oilseed rape yield decline at the landscape scale. Microbiome.

[53] Santos SS (2020). Land use as a driver for protist community structure in soils under agricultural use across Europe. Sci. Total Environ..

[54] Kenmotsu H, Ishikawa M, Nitta T, Hirose Y, Eki T (2021). Distinct community structures of soil nematodes from three ecologically different sites revealed by high-throughput amplicon sequencing of four 18S ribosomal RNA gene regions. PLoS One.

[55] Sapkota R, Nicolaisen M (2015). High-throughput sequencing of nematode communities from total soil DNA extractions. BMC Ecol..

[56] Ablimit R (2022). Altering microbial community for improving soil properties and agricultural sustainability during a 10-year maize-green manure intercropping in Northwest China. J. Environ. Manag..

[57] Hartman K (2018). Cropping practices manipulate abundance patterns of root and soil microbiome members paving the way to smart farming. Microbiome.

[58] Karimi B (2019). Biogeography of soil bacterial networks along a gradient of cropping intensity. Sci. Rep..

[59] Rossmann M (2020). Multitrophic interactions in the rhizosphere microbiome of wheat: From bacteria and fungi to protists. FEMS Microbiol. Ecol..

[60] Ji LF (2020). Effect of organic substitution rates on soil quality and fungal community composition in a tea plantation with long-term fertilization. Biol. Fertil. Soils.

[61] Lee SA (2020). Different types of agricultural land use drive distinct soil bacterial communities. Sci. Rep..

[62] Lewin S, Francioli D, Ulrich A, Kolb S (2021). Crop host signatures reflected by co-association patterns of keystone Bacteria in the rhizosphere microbiota. Environ. Microbiome.

[63] Xiong C (2021). Plant developmental stage drives the differentiation in ecological role of the maize microbiome. Microbiome.

[64] Xie Y (2022). Crop rotation stage has a greater effect than fertilisation on soil microbiome assembly and enzymatic stoichiometry. Sci. Total Environ..

[65] Trochine A (2022). Genomic and proteomic analysis of *Tausonia pullulans* reveals a key role for a GH15 glucoamylase in starch hydrolysis. Appl. Microbiol. Biotechnol..

[66] Carvajal M, Godoy L, Gebauer M, Catrileo D, Albornoz F (2023). Screening for indole-3-acetic acid synthesis and 1-aminocyclopropane-carboxylate deaminase activity in soil yeasts from Chile uncovers *Solicoccozyma aeria* as an effective plant growth promoter. Plant Soil.

[67] Ekelund F, Ronn R, Griffiths BS (2001). Quantitative estimation of flagellate community structure and diversity in soil samples. Protist.

[68] Fiore-Donno AM (2019). Functional traits and spatio-temporal structure of a major group of soil protists (Rhizaria: Cercozoa) in a temperate grassland. Front. Microbiol..

[69] Zhang WL, Lin QM, Li GT, Zhao XR (2022). The ciliate protozoan *Colpoda cucullus* can improve maize growth by transporting soil phosphates. J. Integr. Agric..

[70] Geisen S (2018). Soil protists: A fertile frontier in soil biology research. FEMS Microbiol. Rev..

[71] Huang X (2021). Protists modulate fungal community assembly in paddy soils across climatic zones at the continental scale. Soil Biol. Biochem..

[72] Sikder MM (2021). Benzoxazinoids selectively affect maize root-associated nematode taxa. J. Exp. Bot..

[73] Hirose Y (2020). Investigating algal communities in lacustrine and hydro-terrestrial environments of east antarctica using deep amplicon sequencing. Microorganisms.

[74] Bolyen E (2019). Reproducible, interactive, scalable and extensible microbiome data science using QIIME 2. Nat. Biotechnol..

[75] Martin M (2011). Cutadapt removes adapter sequences from high-throughput sequencing reads. EMBnet.journal.

[76] Callahan BJ (2016). DADA2: High-resolution sample inference from Illumina amplicon data. Nat. Methods.

[77] Rognes T, Flouri T, Nichols B, Quince C, Mahe F (2016). VSEARCH: A versatile open source tool for metagenomics. PeerJ.

[78] Quast C (2013). The SILVA ribosomal RNA gene database project: Improved data processing and web-based tools. Nucleic Acids Res..

[79] McMurdie PJ, Holmes S (2013). phyloseq: An R package for reproducible interactive analysis and graphics of microbiome census data. PLoS One.

[80] Anderson MJ (2001). A new method for non-parametric multivariate analysis of variance. Austral. Ecol..

