# Supplementary material for: Distinct prokaryotic and eukaryotic communities and networks in two agricultural fields of central Japan with different histories of maize–cabbage rotation
Source: Sci Rep. 2023 Sep 18;13:15435. doi: 10.1038/s41598-023-42291-y (PMC10507100; doi:10.1038/s41598-023-42291-y)
Supplement: Supplementary file 1 — Supplementary Information. [file 41598_2023_42291_MOESM1_ESM.pdf]

Supplementary Information

Distinct prokaryotic and eukaryotic communities and networks in two agricultural fields of central Japan with different histories of maize-cabbage rotation

Harutaro Kenmotsu[1], Tomoro Masuma[1], Junya Murakami[1], Yuu Hirose[1,2], and Toshihiko Eki[1,2]

[1]Department of Applied Chemistry and Life Science, Toyohashi University of Technology, 1-1 Hibarigaoka, Tempaku, Toyohashi, Aichi 441-8580, Japan

[2]Research Center for Agrotechnology and Biotechnology, Toyohashi University of Technology, 1-1 Hibarigaoka, Tempaku, Toyohashi, Aichi 441-8580, Japan

Table S1. Comparison and crop rotation-mediated change of relative abundance of eukaryotic and prokaryotic sequence variants (SVs) at family and genus levels and the related information of node SVs

The relative abundance of major famlies and genera was compared between the two fields and the two crops (maize and cabbage) based on Fig. S1, and the results were reported using inequality or equality signs. The changes in relative abundance in each field were summrized and indicated by upward (increase) and downward (decrease) arrows (right arrows: no chande). The node SVs and core nSVs in each family or prokaryotic genus are shown with classification of the corresponding nSVs (see Supplementary Tables S10 and S11 legends). The data from eukaryotic genera were omitted because most of the SVs were unassigned to a genus. Note: Euglyphida order is incorrectly assigned to family in the SILVA database.

Family/genus	Comparison of relative abundance		Change of relative abundance by cropping from maize to cabbage		Node SV (nSV)	Core nSV	Classification of nSVs
	Field_1 vs. Field_2	Maize vs. Cabbage	Field_1	Field_2			
Eukaryotic family							
Mrakiaceae (Basidiomycota)（SV_2)	Field_1 ≪ Field_2	Maize > Cabbage	↓	↓↓	SV_2	SV_2	Common nSV
Mortierellaceae (Mucoromycota)	Field_1 ≪ Field_2	Maize ≧ Cabbage	→	↓	SV_7, 25, 30, 41 (genus *Mortierella*)	SV_7	Field_2-specific nSVs; Common nSV (SV_7)
Filobasidiaceae (Basidiomycota) (SV_4)	Feild_1 ≧ Field_2	Maize ≦ Cabbage	↑	→	SV_4	SV_4	Common nSV
Chaetomiaceae (Ascomycota)	Feild_1 ≦ Field_2	Maize < Cabbage	↑	↑	SV_8, 42	SV_8, 42	Common nSVs
Euglyphida (Cercozoa)	Feild_1 ≦ Field_2	Maize < Cabbage	↑	↑	SV_13, 53, 104, 137	SV_13	Cabbage-specific nSVs; Common nSV (SV_13)
Prokaryotic family							
Sphingomonadaceae (Pseudomonadota)	Feild_1 < Field_2	Maize > Cabbage	↓↓	↓↓	SV_1	SV_1	Common nSV
Rhodanobacteraceae (Pseudomonadota)	Feild_1 > Field_2	Maize ≧ Cabbage	↓↓	↓	SV_8, 24	SV_8	Maize-specific nSVs
Chitinophagaceae (Bacteroidota)	Feild_1 ≒ Field_2	Maize > Cabbage	↓	↓	SV_117		Maize-specific nSVs
Chthoniobacteraceae (Verrucomicrobiota)	Feild_1 ≦ Field_2	Maize ≧ Cabbage	→	↓			
Gemmatimonadaceae (Gemmatimonadota)	Feild_1 ≒ Field_2	Maize ≒ Cabbage	↑	→	SV_70, 93; SV_143; SV_159	SV_143	Cabbage-specific nSVs (SV_70, 93); Field_1-specific nSV (SV_143); Maize-specific nSVs (SV_159)
Vicinamibacteraceae (Acidobacteriota)	Feild_1 ≒ Field_2	Maize ≦ Cabbage	↑	↑	SV_11, 243	SV_243	Cabbage-specific nSVs
Nitrosomonadaceae (Pseudomonadota)	Feild_1 ≒ Field_2	Maize < Cabbage	→	↑	SV_142, 279		Feild_2-specific nSVs
Pyrinomonadaceae (Acidobacteriota)	Feild_1 < Field_2	Maize ≦ Cabbage	→	↑	SV_15, 171, 221, 229, 268	SV_15	Feild_2-specific nSVs
Pedosphaeraceae (Verrucomicrobiota)	Feild_1 ≦ Field_2	Maize ≒ Cabbage	→	→			
WD2101_soil_group [Tepidisphaeraceae](Planctomycetota)	Feild_1 < Field_2	Maize < Cabbage	→	→	SV_186	SV_186	Cabbage-specific nSV
Prokaryotic genus							
Sphingomonas (Sphingomonadaceae, Pseudomonadota)	Feild_1 < Field_2	Maize > Cabbage	↓↓	↓↓	SV_1; SV_61	SV_1; SV_61	Common nSV; Feild_2-specific nSV
Rhodanobacter (Rhodanobacteraceae, Pseudomonadota)	Feild_1 > Field_2	Maize > Cabbage	↓↓	↓	SV_8, 24	SV_8	Maize-specific nSVs
Nocardioides (Nocardioidaceae, Actinobacteriota)	Feild_1 > Field_2	Maize > Cabbage	↓	↓	SV_121		Maize-specific nSVs
RB41 (Pyrinomonadaceae, Acidobacteriota)	Feild_1 < Field_2	Maize ≦ Cabbage	→	↑↑	SV_15, 171, 221, 229, 268	SV_15	Feild_2-specific nSVs
Acidibacter (Pseudomonadota)	Feild_1 ≧ Field_2	Maize ≦ Cabbage	→	↑	SV_16		Common nSV
Gemmatimonas (Gemmatimonadaceae, Gemmatimonadota)	Feild_1 ≦ Field_2	Maize ≒ Cabbage	→	→	SV_159; SV_189	SV_189	Maize-specific nSV; Feild_2-specific nSV

Table S2. Tukey-Kramer test for asseccing difference of alpha diversity between two sample groups.

Difference of Shannon indexes between two sample groups from diferent crops, fields and growth stages were investigated by Tukey-Kramer test. P adj: ajusted *p* -value. Less than 0.05 of p adj was considered significantly different and indicated by yellow color. Abbreviations of sample groups are shown as "Field_1_maize" (maize-cultivated field_1).

Eukaryotic alpha diversity of four sample groups

Sample group 1	Sample group 2	diff	lwr	upr	p adj
Field_1_maize	Field_1_cabbage	-0.43102	-0.82807	-0.03397	0.02924
Field_2_cabbage	Field_1_cabbage	0.19958	-0.19747	0.59663	0.53167
Field_2_maize	Field_1_cabbage	-0.05542	-0.45247	0.34163	0.98124
Field_2_cabbage	Field_1_maize	0.63061	0.23356	1.02766	0.00082
Field_2_maize	Field_1_maize	0.37560	-0.02145	0.77265	0.06907
Field_2_maize	Field_2_cabbage	-0.25501	-0.65206	0.14204	0.32040

Prokaryotic alpha diversity of four sample groups

Sample group 1	Sample group 2	diff	lwr	upr	p adj
Field_1_maize	Field_1_cabbage	0.06491	-0.17439	0.30420	0.88232
Field_2_cabbage	Field_1_cabbage	-0.04661	-0.28590	0.19268	0.95173
Field_2_maize	Field_1_cabbage	-0.05503	-0.29433	0.18426	0.92391
Field_2_cabbage	Field_1_maize	-0.11152	-0.35081	0.12778	0.59269
Field_2_maize	Field_1_maize	-0.11994	-0.35923	0.11935	0.53405
Field_2_maize	Field_2_cabbage	-0.00842	-0.24772	0.23087	0.99968

Eukaryotic alpha diversity of four sample groups at three stages

Sample group 1	Sample group 2	diff	lwr	upr	p adj
Field_1_maize_early	Field_1_cabbage_early	-0.57730	-1.38145	0.22686	0.33794
Field_2_cabbage_early	Field_1_cabbage_early	-0.19392	-0.99808	0.61024	0.99887
Feild_2_maize_early	Field_1_cabbage_early	-0.20847	-1.01263	0.59568	0.99786
Field_1_cabbage_middle	Field_1_cabbage_early	-0.32612	-1.13028	0.47804	0.93717
Field_1_maize_middle	Field_1_cabbage_early	-0.55524	-1.35940	0.24891	0.39069
Field_2_cabbage_middle	Field_1_cabbage_early	-0.24538	-1.04953	0.55878	0.99171
Field_2_maize_middle	Field_1_cabbage_early	-0.52767	-1.33182	0.27649	0.46212
Field_1_cabbage_late	Field_1_cabbage_early	-0.38562	-1.18978	0.41853	0.83812
Field_1_maize_late	Field_1_cabbage_early	-0.87227	-1.67643	-0.06812	0.02548
Field_2_cabbage_late	Field_1_cabbage_early	0.32631	-0.47784	1.13047	0.93694
Field_2_maize_late	Field_1_cabbage_early	-0.14187	-0.94603	0.66228	0.99994
Field_2_cabbage_early	Field_1_maize_early	0.38338	-0.42078	1.18753	0.84282
Field_2_maize_early	Field_1_maize_early	0.36883	-0.43533	1.17298	0.87159
Field_1_cabbage_middle	Field_1_maize_early	0.25118	-0.55298	1.05533	0.99004
Field_1_maize_middle	Field_1_maize_early	0.02206	-0.78210	0.82621	1.00000
Field_2_cabbage_middle	Field_1_maize_early	0.33192	-0.47223	1.13608	0.92993
Field_2_maize_middle	Field_1_maize_early	0.04963	-0.75452	0.85379	1.00000
Field_1_cabbage_late	Field_1_maize_early	0.19168	-0.61248	0.99583	0.99898
Field_1_maize_late	Field_1_maize_early	-0.29497	-1.09913	0.50918	0.96750
Field_2_cabbage_late	Field_1_maize_early	0.90361	0.09945	1.70777	0.01851
Field_2_maize_late	Field_1_maize_early	0.43543	-0.36873	1.23958	0.71796
Field_2_maize_early	Field_2_cabbage_early	-0.01455	-0.81871	0.78960	1.00000
Field_1_cabbage_middle	Field_2_cabbage_early	-0.13220	-0.93636	0.67196	0.99997
Field_1_maize_middle	Field_2_cabbage_early	-0.36132	-1.16548	0.44283	0.88517
Field_2_cabbage_middle	Field_2_cabbage_early	-0.05146	-0.85561	0.75270	1.00000
Field_2_maize_middle	Field_2_cabbage_early	-0.33375	-1.13790	0.47041	0.92755
Field_1_cabbage_late	Field_2_cabbage_early	-0.19170	-0.99586	0.61245	0.99898

Field_1_maize_late	Field_2_cabbage_early	-0.67835	-1.48251	0.12580	0.15646
Field_2_cabbage_late	Field_2_cabbage_early	0.52023	-0.28392	1.32439	0.48222
Field_2_maize_late	Field_2_cabbage_early	0.05205	-0.75211	0.85620	1.00000
Field_1_cabbage_middle	Field_2_maize_early	-0.11765	-0.92180	0.68651	0.99999
Field_1_maize_middle	Field_2_maize_early	-0.34677	-1.15093	0.45739	0.90903
Field_2_cabbage_middle	Field_2_maize_early	-0.03690	-0.84106	0.76725	1.00000
Field_2_maize_middle	Field_2_maize_early	-0.31919	-1.12335	0.48496	0.94513
Field_1_cabbage_late	Field_2_maize_early	-0.17715	-0.98130	0.62701	0.99950
Field_1_maize_late	Field_2_maize_early	-0.66380	-1.46795	0.14036	0.17647
Field_2_cabbage_late	Field_2_maize_early	0.53478	-0.26937	1.33894	0.44318
Field_2_maize_late	Field_2_maize_early	0.06660	-0.73756	0.87076	1.00000
Field_1_maize_middle	Field_1_cabbage_middle	-0.22912	-1.03328	0.57503	0.99524
Field_2_cabbage_middle	Field_1_cabbage_middle	0.08074	-0.72341	0.88490	1.00000
Field_2_maize_middle	Field_1_cabbage_middle	-0.20155	-1.00570	0.60261	0.99841
Field_1_cabbage_late	Field_1_cabbage_middle	-0.05950	-0.86366	0.74465	1.00000
Field_1_maize_late	Field_1_cabbage_middle	-0.54615	-1.35031	0.25800	0.41363
Field_2_cabbage_late	Field_1_cabbage_middle	0.65243	-0.15173	1.45659	0.19347
Field_2_maize_late	Field_1_cabbage_middle	0.18425	-0.61991	0.98840	0.99929
Field_2_cabbage_middle	Field_1_maize_middle	0.30987	-0.49429	1.11402	0.95472
Field_2_maize_middle	Field_1_maize_middle	0.02758	-0.77658	0.83173	1.00000
Field_1_cabbage_late	Field_1_maize_middle	0.16962	-0.63453	0.97378	0.99967
Field_1_maize_late	Field_1_maize_middle	-0.31703	-1.12118	0.48713	0.94747
Field_2_cabbage_late	Field_1_maize_middle	0.88155	0.07740	1.68571	0.02319
Field_2_maize_late	Field_1_maize_middle	0.41337	-0.39079	1.21753	0.77449
Field_2_maize_middle	Field_2_cabbage_middle	-0.28229	-1.08644	0.52187	0.97613
Field_1_cabbage_late	Field_2_cabbage_middle	-0.14024	-0.94440	0.66391	0.99995
Field_1_maize_late	Field_2_cabbage_middle	-0.62689	-1.43105	0.17726	0.23626
Field_2_cabbage_late	Field_2_cabbage_middle	0.57169	-0.23247	1.37584	0.35095
Field_2_maize_late	Field_2_cabbage_middle	0.10350	-0.70065	0.90766	1.00000
Field_1_cabbage_late	Field_2_maize_middle	0.14204	-0.66211	0.94620	0.99994
Field_1_maize_late	Field_2_maize_middle	-0.34461	-1.14876	0.45955	0.91229
Field_2_cabbage_late	Field_2_maize_middle	0.85398	0.04982	1.65813	0.03063
Field_2_maize_late	Field_2_maize_middle	0.38579	-0.41836	1.18995	0.83775
Field_1_maize_late	Field_1_cabbage_late	-0.48665	-1.29081	0.31751	0.57579
Field_2_cabbage_late	Field_1_cabbage_late	0.71193	-0.09222	1.51609	0.11730
Field_2_maize_late	Field_1_cabbage_late	0.24375	-0.56041	1.04790	0.99214
Field_2_cabbage_late	Field_1_maize_late	1.19858	0.39443	2.00274	0.00080
Field_2_maize_late	Field_1_maize_late	0.73040	-0.07376	1.53455	0.09956
Field_2_maize_late	Field_2_cabbage_late	-0.46818	-1.27234	0.33597	0.62791

Prokaryotic alpha diversity of four sample groups at three stages

Sample group 1	Sample group 2	diff	lwr	upr	p adj
Field_1_maize_early	Field_1_cabbage_early	-0.10953	-0.59035	0.37128	0.99932
Field_2_cabbage_early	Field_1_cabbage_early	-0.19268	-0.67349	0.28813	0.94170
Feild_2_maize_early	Field_1_cabbage_early	-0.45252	-0.93334	0.02829	0.07823
Field_1_cabbage_middle	Field_1_cabbage_early	-0.20168	-0.68249	0.27913	0.92275
Field_1_maize_middle	Field_1_cabbage_early	-0.02033	-0.50114	0.46048	1.00000
Field_2_cabbage_middle	Field_1_cabbage_early	-0.14908	-0.62989	0.33174	0.99060
Field_2_maize_middle	Field_1_cabbage_early	-0.08178	-0.56259	0.39903	0.99996
Field_1_cabbage_late	Field_1_cabbage_early	-0.12755	-0.60837	0.35326	0.99739
Field_1_maize_late	Field_1_cabbage_early	-0.00465	-0.48546	0.47616	1.00000
Field_2_cabbage_late	Field_1_cabbage_early	-0.12731	-0.60812	0.35350	0.99743
Field_2_maize_late	Field_1_cabbage_early	0.03997	-0.44084	0.52078	1.00000
Field_2_cabbage_early	Field_1_maize_early	-0.08314	-0.56396	0.39767	0.99995

Field_2_maize_early	Field_1_maize_early	-0.34299	-0.82380	0.13782	0.34637
Field_1_cabbage_middle	Field_1_maize_early	-0.09215	-0.57296	0.38867	0.99987
Field_1_maize_middle	Field_1_maize_early	0.08920	-0.39161	0.57002	0.99990
Field_2_cabbage_middle	Field_1_maize_early	-0.03954	-0.52035	0.44127	1.00000
Field_2_maize_middle	Field_1_maize_early	0.02775	-0.45306	0.50857	1.00000
Field_1_cabbage_late	Field_1_maize_early	-0.01802	-0.49883	0.46279	1.00000
Field_1_maize_late	Field_1_maize_early	0.10488	-0.37593	0.58569	0.99955
Field_2_cabbage_late	Field_1_maize_early	-0.01778	-0.49859	0.46303	1.00000
Field_2_maize_late	Field_1_maize_early	0.14950	-0.33131	0.63032	0.99039
Field_2_maize_early	Field_2_cabbage_early	-0.25985	-0.74066	0.22097	0.72017
Field_1_cabbage_middle	Field_2_cabbage_early	-0.00900	-0.48981	0.47181	1.00000
Field_1_maize_middle	Field_2_cabbage_early	0.17235	-0.30846	0.65316	0.97232
Field_2_cabbage_middle	Field_2_cabbage_early	0.04360	-0.43721	0.52441	1.00000
Field_2_maize_middle	Field_2_cabbage_early	0.11090	-0.36991	0.59171	0.99924
Field_1_cabbage_late	Field_2_cabbage_early	0.06512	-0.41569	0.54594	1.00000
Field_1_maize_late	Field_2_cabbage_early	0.18803	-0.29278	0.66884	0.95014
Field_2_cabbage_late	Field_2_cabbage_early	0.06537	-0.41544	0.54618	1.00000
Field_2_maize_late	Field_2_cabbage_early	0.23265	-0.24816	0.71346	0.83067
Field_1_cabbage_middle	Field_2_maize_early	0.25085	-0.22997	0.73166	0.75914
Field_1_maize_middle	Field_2_maize_early	0.43219	-0.04862	0.91301	0.10652
Field_2_cabbage_middle	Field_2_maize_early	0.30345	-0.17736	0.78426	0.51722
Field_2_maize_middle	Field_2_maize_early	0.37074	-0.11007	0.85156	0.24879
Field_1_cabbage_late	Field_2_maize_early	0.32497	-0.15584	0.80578	0.42041
Field_1_maize_late	Field_2_maize_early	0.44787	-0.03294	0.92869	0.08404
Field_2_cabbage_late	Field_2_maize_early	0.32521	-0.15560	0.80603	0.41936
Field_2_maize_late	Field_2_maize_early	0.49249	0.01168	0.97331	0.04134
Field_1_maize_middle	Field_1_cabbage_middle	0.18135	-0.29946	0.66216	0.96072
Field_2_cabbage_middle	Field_1_cabbage_middle	0.05260	-0.42821	0.53342	1.00000
Field_2_maize_middle	Field_1_cabbage_middle	0.11990	-0.36091	0.60071	0.99847
Field_1_cabbage_late	Field_1_cabbage_middle	0.07412	-0.40669	0.55494	0.99998
Field_1_maize_late	Field_1_cabbage_middle	0.19703	-0.28378	0.67784	0.93298
Field_2_cabbage_late	Field_1_cabbage_middle	0.07437	-0.40644	0.55518	0.99998
Field_2_maize_late	Field_1_cabbage_middle	0.24165	-0.23916	0.72246	0.79666
Field_2_cabbage_middle	Field_1_maize_middle	-0.12875	-0.60956	0.35207	0.99717
Field_2_maize_middle	Field_1_maize_middle	-0.06145	-0.54226	0.41936	1.00000
Field_1_cabbage_late	Field_1_maize_middle	-0.10722	-0.58804	0.37359	0.99944
Field_1_maize_late	Field_1_maize_middle	0.01568	-0.46513	0.49649	1.00000
Field_2_cabbage_late	Field_1_maize_middle	-0.10698	-0.58779	0.37383	0.99945
Field_2_maize_late	Field_1_maize_middle	0.06030	-0.42051	0.54111	1.00000
Field_2_maize_middle	Field_2_cabbage_middle	0.06730	-0.41352	0.54811	0.99999
Field_1_cabbage_late	Field_2_cabbage_middle	0.02152	-0.45929	0.50233	1.00000
Field_1_maize_late	Field_2_cabbage_middle	0.14442	-0.33639	0.62524	0.99269
Field_2_cabbage_late	Field_2_cabbage_middle	0.02176	-0.45905	0.50258	1.00000
Field_2_maize_late	Field_2_cabbage_middle	0.18905	-0.29177	0.66986	0.94837
Field_1_cabbage_late	Field_2_maize_middle	-0.04578	-0.52659	0.43504	1.00000
Field_1_maize_late	Field_2_maize_middle	0.07713	-0.40368	0.55794	0.99998
Field_2_cabbage_late	Field_2_maize_middle	-0.04553	-0.52634	0.43528	1.00000
Field_2_maize_late	Field_2_maize_middle	0.12175	-0.35906	0.60256	0.99825
Field_1_maize_late	Field_1_cabbage_late	0.12290	-0.35791	0.60372	0.99811
Field_2_cabbage_late	Field_1_cabbage_late	0.00024	-0.48057	0.48106	1.00000
Field_2_maize_late	Field_1_cabbage_late	0.16752	-0.31329	0.64834	0.97738
Field_2_cabbage_late	Field_1_maize_late	-0.12266	-0.60347	0.35815	0.99814
Field_2_maize_late	Field_1_maize_late	0.04462	-0.43619	0.52543	1.00000
Field_2_maize_late	Field_2_cabbage_late	0.16728	-0.31353	0.64809	0.97762

Table S3. Soil chemical parameter data.

Mean values and the standard deviations of indicated soil chemical parameters in each sample group are shown in each columns. Each data was derived from three independent soil samples. EC: electric conductivity (dS/m) (1:5); pH: pH in water (1:2.5); NH4_N: ammonium nitrogen (mg/100 g soil); NO3_N: nitrate nitrogen; CEC: cation-exchange capacity (me/100 g soil); Tr_P2O5: available phosphorus (mg/100 g soil); CaO: exchangeable calcium (mg/100 g soil); MgO: exchangeable magnesium (mg/100 g soil); K2O: exchangeable potassium (mg/100 g soil); Humus: humus content (%); WC: water content (see Materials and methods). Abbreviations of sample groups are described in the Supplementary Table S2 legend.

Mean value

Sample group	Growth stage	EC	pH_H2O	NH4_N	NO3_N	Tr_P2O5	CEC	CaO	MgO	K2O	Humus	WC
Field_1_maize	Early	0.48	5.86	0.77	15.7	68.0	12.1	200	50.0	66.0	2.17	0.09
	Middle	0.16	6.17	1.40	2.47	89.3	12.3	157	36.0	50.0	2.40	0.07
	Late	0.14	6.23	0.73	2.93	86.3	12.3	160	35.7	51.3	2.33	0.10
Field_1_cabbage	Early	0.13	6.63	0.33	2.40	94.3	10.4	114	29.0	51.0	1.97	0.06
	Middle	0.20	6.30	0.73	3.63	72.0	15.0	143	39.0	57.3	1.93	0.05
	Late	0.08	6.62	0.60	0.30	62.3	12.3	137	34.7	46.0	2.07	0.07
Field_2_maize	Early	0.30	6.01	1.03	6.23	273	18.0	270	63.7	86.7	1.63	0.19
	Middle	0.26	5.87	1.33	9.40	290	20.0	283	61.3	95.3	1.73	0.14
	Late	0.12	6.03	0.47	2.33	290	19.0	283	58.7	76.0	1.73	0.18
Field_2_cabbage	Early	0.19	6.73	0.37	2.17	320	19.3	303	62.3	86.7	1.50	0.13
	Middle	0.18	6.54	0.80	2.67	277	21.7	320	72.7	91.0	1.43	0.09
	Late	0.10	6.63	1.10	0.43	280	21.0	307	69.3	89.3	1.67	0.12

Standard deviation

Sample group	Growth stage	EC	pH_H2O	NH4_N	NO3_N	Tr_P2O5	CEC	CaO	MgO	K2O	Humus	WC
Field_1_maize	Early	0.14	0.15	0.17	4.03	8.64	2.02	40.8	9.42	2.16	0.40	0.01
	Middle	0.02	0.03	0.71	0.63	8.22	0.47	9.43	2.94	4.55	0.08	0.01
	Late	0.06	0.33	0.17	2.05	1.25	0.47	16.3	4.11	4.50	0.17	0.01
Field_1_cabbage	Early	0.01	0.12	0.05	0.14	11.4	0.85	16.1	3.74	8.64	0.05	0.01
	Middle	0.07	0.27	0.09	1.41	2.16	0.00	12.5	1.63	1.25	0.12	0.00
	Late	0.01	0.11	0.24	0.08	8.22	0.47	17.0	0.47	4.32	0.25	0.00
Field_2_maize	Early	0.02	0.06	0.05	0.05	14.1	0.94	12.5	2.36	3.68	0.08	0.01
	Middle	0.03	0.06	0.36	1.52	4.71	0.94	8.16	1.70	6.48	0.09	0.00
	Late	0.01	0.09	0.22	0.26	21.6	1.41	33.0	5.25	11.1	0.05	0.01
Field_2_cabbage	Early	0.02	0.05	0.21	0.62	12.5	0.00	14.1	1.70	1.25	0.05	0.00
	Middle	0.08	0.07	0.33	2.26	8.16	1.63	34.0	7.76	11.5	0.05	0.01
	Late	0.02	0.03	0.09	0.75	8.16	0.82	20.5	1.70	2.45	0.05	0.01

Table S4. Eukaryotic and prokaryotic families and sequence variants closely associated with soil chemical parameters.

Eukaryotic and prokaryotic families and sequence variants (SVs) closely associated with humus, pH, nitrate nitrogen, nutrient ions/phosphorus, and water content, are identified in RDA plots of the Supplementary Fig. S4. Family names and their phyla are in parentheses for associated families, and the SVs, their phyla, families and information of node SVs are shown after sorting by phylum and family. NA: Unassigned phylum or family. Taxa are derived from the SILVA database (note: some of taxa such as Euglyphida order and Colpodea class are incorrectly assigned to family in the SILVA database). RDA was performed using top 350 SVs. Abbreviations of common, cabbage-, maize-, field_2-specific are common nSV, and cabbage-, maize-, field_2-specific nSVs, respectively. Data in yellow-colored cells are referred in the text.

Humus

Associated eukaryotic family				Associated prokaryotic family			
Mucoraceae (Mucoromycota)				Promicromonosporaceae (Actinomycetota)			
Pleosporaceae (Ascomycota)				Micromonosporaceae (Actinomycetota)			
**Eukaryotic SVs**	**Phylum**	**Family**	**Node SV**	**Prokaryotic SVs**	**Phylum**	**Family**	**Node SV**
SV_119	Arthropoda	NA		SV_41	Actinomycetota	Nocardioidaceae	
SV_5	Ascomycota	NA	Common	SV_103	Actinomycetota	Geodermatophilaceae	Cabbage-specific
SV_209	Ascomycota	NA		SV_131	Actinomycetota	Micromonosporaceae	
SV_295	Cercozoa	Euglyphida		SV_170	Chloroflexota	NA	Field_2-specific
SV_10	Mucoromycota	Mucoraceae		SV_309	Chloroflexota	NA	
SV_148	Tubulinea	NA		SV_45	Pseudomonadota	Methyloligellaceae	Common
				SV_35	Pseudomonadota	NA	

pH

Associated eukaryotic family				Associated prokaryotic family			
Euglyphida (Cercozoa)				KF-JG30-B3 (Environmental sample)			
Oligohymenophorea (Ciliophora)				Reyranellaceae (Pseudomonadota)			
				Ferrovibrionaceae (Pseudomonadota?)			
				Xanthobacteraceae (Pseudomonadota)			
				Rhodospirillaceae (Pseudomonadota)			
**Eukaryotic SVs**	**Phylum**	**Family**	**Node SV**	**Prokaryotic SVs**	**Phylum**	**Family**	**Node SV**
SV_170	Annelida	NA		SV_343	Acidobacteriota	Vicinamibacteraceae	
SV_187	Basidiomycota	Ustilaginaceae		SV_307	Actinomycetota	NA	
SV_13	Cercozoa	Euglyphida	Common	SV_253	Candidatus Latescibacteria	NA	
SV_51	Cercozoa	Euglyphida		SV_153	Myxococcota	BIrii41	Cabbage-specific
SV_104	Cercozoa	Euglyphida	Cabbage-specific	SV_340	Pseudomonadota	Xanthobacteraceae	Cabbage-specific
SV_137	Cercozoa	Euglyphida	Cabbage-specific	SV_182	Verrucomicrobiota	Opitutaceae	
SV_54	Cercozoa	NA		SV_37	Verrucomicrobiota	Pedosphaeraceae	Cabbage-specific
SV_175	Cercozoa	NA	Cabbage-specific				
SV_223	Cercozoa	NA	Cabbage-specific				
SV_243	Ciliophora	Colpodea	Cabbage-specific				
SV_248	Nematoda	NA					
SV_3	Streptophyta	NA (cabbage)					

Water contents

Associated eukaryotic family				Associated prokaryotic family			
Glomerellaceae (Ascomycota)				Sphingomonadaceae (Pseudomonadota)			
Rhizophydiaceae (Chytridiomycota)				Latescibacteraceae (Candidatus Latescibacteria)			
				Verrucomicrobiaceae (Verrucomicrobiota)			
**Eukaryotic SVs**	**Phylum**	**Family**	**Node SV**	**Prokaryotic SVs**	**Phylum**	**Family**	**Node SV**
SV_112	Ascomycota	Glomerellaceae	Field_2-specific	SV_275	Bacteroidota	Chitinophagaceae	Field_2-specific
SV_313	Ascomycota	NA		SV_74	Gemmatimonadota	Gemmatimonadaceae	Field_2-specific
SV_2	Basidiomycota	Mrakiaceae	Common	SV_195	Pseudomonadota	Beijerinckiaceae	
SV_140	Ciliophora	Colpodea		SV_148	Pseudomonadota	Xanthomonadaceae	
SV_145	Nematoda	NA		SV_248	Pseudomonadota	NA	Field_2-specific
SV_64	Oomycota	NA		SV_206	Verrucomicrobiota	Chthoniobacteraceae	Field_2-specific
SV_329	NA (Stramenopile	NA					

Nitrate nitrogen

Associated eukaryotic family				Associated prokaryotic family			
Cladosporiaceae (Ascomycota)				Nocardiaceae (Actinomycetota)			
Didymellaceae (Ascomycota)				Kineosporiaceae (Actinomycetota)			
Plectosphaerellaceae (Ascomycota)				Rhodocyclaceae (Pseudomonadota)			
				Alcaligenaceae (Pseudomonadota)			
				Cellvibrionaceae (Pseudomonadota)			
**Eukaryotic SVs**	**Phylum**	**Family**	**Node SV**	**Prokaryotic SVs**	**Phylum**	**Family**	**Node SV**
SV_134	Arthropoda	NA		SV_92	Actinomycetota	Frankiaceae	
SV_147	Ascomycota	Cladosporiaceae		SV_181	Actinomycetota	Micrococcaceae	
SV_27	Ascomycota	Didymellaceae		SV_350	Actinomycetota	Micrococcaceae	
SV_37	Ascomycota	Plectosphaerellaceae	Maize-specific	SV_284	Actinomycetota	Nocardioidaceae	
SV_173	Ascomycota	Plectosphaerellaceae		SV_299	Actinomycetota	Nocardioidaceae	
SV_94	Ascomycota	Pleosporaceae	Maize-specific	SV_117	Bacteroidota	Chitinophagaceae	Maize-specific
SV_204	Ascomycota	Pleosporaceae		SV_311	Gemmatimonadota	Gemmatimonadaceae	
SV_66	Cercozoa	Rhizaspididae		SV_116	Nitrospirota	Nitrospiraceae	
SV_203	Cercozoa	NA	Maize-specific	SV_277	Pseudomonadota	Micropepsaceae	
SV_281	Ciliophora	Oligohymenophorea		SV_224	Pseudomonadota	Pseudomonadaceae	
SV_26	Nematoda	NA		SV_24	Pseudomonadota	Rhodanobacteraceae	Maize-specific
SV_31	Nematoda	NA		SV_73	Pseudomonadota	Sphingomonadaceae	Field_2-specific
SV_334	Nematoda	NA		SV_157	Pseudomonadota	Sphingomonadaceae	Field_2-specific
SV_1	Streptophyta	NA (maize)		SV_339	Pseudomonadota	Sphingomonadaceae	
				SV_336	Pseudomonadota	Sphingomonadaceae	
				SV_23	Pseudomonadota	Xanthobacteraceae	Common

Nutrient ions and phosphorus

Associated eukaryotic family				Associated prokaryotic family			
Ascodesmidaceae (Ascomycota)				Planococcaceae (Bacillota)			

Trichosporonaceae (Basidiomycota)<br>Pyronemataceae (Ascomycota)<br>Mortierellaceae (Mucoromycota)				Syntrophobacteraceae (Thermodesulfobacteriota)<br>Erysipelotrichaceae (Bacillota)<br>Bacillaceae (Bacillota)<br>Chthoniobacteraceae (Verrucomicrobiota)<br>Comamonadaceae (Pseudomonadota)<br>Pedosphaeraceae (Verrucomicrobiota)			
**Eukaryotic SVs**	**Phylum**	**Family**	**Node SV**	**Prokaryotic SVs**	**Phylum**	**Family**	**Node SV**
SV_73	Ascomycota	Ascodesmidaceae	Field_2-specific	SV_203	Acidobacteriota	Blastocatellaceae	Field_2-specific
SV_266	Ascomycota	Chaetomiaceae		SV_323	Acidobacteriota	NA	
SV_44	Ascomycota	NA	Field_2-specific	SV_364	Acidobacteriota	NA	
SV_127	Ascomycota	NA	Field_2-specific	SV_221	Acidobacteriota	Pyrinomonadaceae	Field_2-specific
SV_274	Ascomycota	NA		SV_268	Acidobacteriota	Pyrinomonadaceae	Field_2-specific
SV_96	Ascomycota	Pyronemataceae	Field_2-specific	SV_229	Acidobacteriota	Pyrinomonadaceae	Field_2-specific
SV_260	Ascomycota	Sympoventuriaceae		SV_171	Acidobacteriota	Pyrinomonadaceae	Field_2-specific
SV_142	Cercozoa	NA	Field_2-specific	SV_3	Bacillota	Bacillaceae	Field_2-specific
SV_156	Cercozoa	NA		SV_202	Candidatus Latescibacteria	NA	
SV_276	Cercozoa	NA		SV_254	Gemmatimonadota	Gemmatimonadaceae	
SV_191	NA (fungi)	NA		SV_310	Gemmatimonadota	Gemmatimonadaceae	Field_2-specific
				SV_189	Gemmatimonadota	Gemmatimonadaceae	Field_2-specific
				SV_191	Planctomycetota	WD2101_soil_group	
				SV_313	Planctomycetota	WD2101_soil_group	
				SV_272	Pseudomonadota	Comamonadaceae	
				SV_305	Pseudomonadota	Methyloligellaceae	
				SV_18	Pseudomonadota	SC-I-84	
				SV_22	Pseudomonadota	SC-I-84	
				SV_288	Pseudomonadota	SC-I-84	
				SV_78	Pseudomonadota	Sphingomonadaceae	Field_2-specific
				SV_279	Pseudomonadota	TRA3-20	Field_2-specific
				SV_263	Verrucomicrobiota	Chthoniobacteraceae	Field_2-specific
				SV_322	Verrucomicrobiota	Chthoniobacteraceae	
				SV_69	Verrucomicrobiota	Pedosphaeraceae	
				SV_271	Verrucomicrobiota	Pedosphaeraceae	

Table S6. Summary of eukaryotic and prokaryotic networks in the four sample gruops.

Numbers of clusters, links, and node sequence variants (nSVs) of the network in the four sample groups are shown. Max cluster size indicates the total number of nSVs belonging to the largest cluster in the network. Abbreviations of sample groups are described in the legend for Supplementary Table S2.

	**Sample groups**	**Cluster numbers**	**Link numbers**	**nSV numbers**	**Max cluster size**
Eukaryotic networks	Field_1_maize	11	86	62	33
	Field_1_cabbage	23	285	121	60
	Field_2_maize	10	359	84	56
	Field_2_cabbage	28	628	170	97
Prokaryotic networks	Field_1_maize	19	127	102	19
	Field_1_cabbage	25	1889	352	214
	Field_2_maize	10	1387	234	203
	Field_2_cabbage	19	3363	402	292

Table S7. Phyla of the eukaryotic and prokarytic node sequence variants in the four sample groups.

The number of node sequence variants (nSVs) derived from eukaryotic and prokaryotic phyla (upper and lower tables, respectively) are shown for each sample group with the percentage of the total nSVs indicated in parenthesis. Data in yellow-colored cells are referred to in the text. Abbreviations of sample groups are described in the legend for Supplementary Table S2.

Eukaryotic phyla	Field_1_maize	Field_1_cabbage	Field_2_maize	Field_2_cabbage
Annelida	0 (0.0)	0 (0.0)	0 (0.0)	2 (1.2)
Apicomplexa	0 (0.0)	0 (0.0)	0 (0.0)	1 (0.6)
Arthropoda	3 (4.8)	3 (2.5)	1 (1.2)	5 (2.9)
Ascomycota	16 (25.8)	27 (22.3)	26 (31.3)	34 (19.9)
Basidiomycota	5 (8.1)	6 (5.0)	5 (6.0)	6 (3.5)
Cercozoa	12 (19.4)	42 (34.7)	25 (30.1)	52 (30.4)
Chlorophyta	0 (0.0)	0 (0.0)	1 (1.2)	0 (0.0)
Chytridiomycota	1 (1.6)	2 (1.7)	3 (3.6)	3 (1.8)
Ciliophora	6 (9.7)	11 (9.1)	4 (4.8)	14 (8.2)
Cryptomycota	0 (0.0)	1 (0.8)	0 (0.0)	0 (0.0)
Mucoromycota	2 (3.2)	4 (3.3)	5 (6.0)	9 (5.3)
Nematoda	4 (6.5)	6 (5.0)	4 (4.8)	9 (5.3)
Oomycota	2 (3.2)	1 (0.8)	2 (2.4)	1 (0.6)
Rotifera	0 (0.0)	0 (0.0)	0 (0.0)	1 (0.6)
Streptophyta	0 (0.0)	0 (0.0)	0 (0.0)	1 (0.6)
Tardigrada	0 (0.0)	1 (0.8)	1 (1.2)	0 (0.0)
Tubulinea	2 (3.2)	2 (1.7)	0 (0.0)	7 (4.1)
NA	9 (14.5)	15 (12.4)	7 (8.4)	25 (14.6)
Total	62 (100.0)	121 (100.0)	84 (100.0)	170 (100.0)

Prokaryotic phyla	Field_1_maize	Field_1_cabbage	Field_2_maize	Field_2_cabbage
Acidobacteriota corrig.	7 (6.9)	65 (18.7)	38 (16.2)	79 (19.7)
Actinomycetota corrig.	30 (29.4)	70 (20.2)	21 (9.0)	31 (7.7)
Armatimonadota corrig.	0 (0.0)	3 (0.9)	1 (0.4)	3 (0.7)
Bacillota corrig.	2 (2.0)	4 (1.2)	4 (1.7)	6 (1.5)
Bacteroidota corrig.	9 (8.8)	6 (1.7)	18 (7.7)	31 (7.7)
Bdellovibrionota	0 (0.0)	0 (0.0)	0 (0.0)	1 (0.2)
Candidatus Dadabacteria	0 (0.0)	1 (0.3)	0 (0.0)	0 (0.0)
Candidate division NC10	0 (0.0)	4 (1.2)	2 (0.9)	4 (1.0)
Candidatus Latescibacteria	0 (0.0)	1 (0.3)	4 (1.7)	5 (1.2)
Chloroflexota corrig.	4 (3.9)	10 (2.9)	9 (3.8)	12 (3.0)
Deinococcota corrig.	0 (0.0)	0 (0.0)	0 (0.0)	1 (0.2)
Elusimicrobiota corrig.	0 (0.0)	1 (0.3)	0 (0.0)	0 (0.0)
Fibrobacterota corrig.	0 (0.0)	1 (0.3)	0 (0.0)	0 (0.0)
Gemmatimonadota corrig.	3 (2.9)	34 (9.8)	22 (9.4)	36 (9.0)
Myxococcota	1 (1.0)	11 (3.2)	6 (2.6)	20 (5.0)
Nitrospirota corrig.	0 (0.0)	3 (0.9)	3 (1.3)	3 (0.7)
Patescibacteria group (clade)	0 (0.0)	2 (0.6)	0 (0.0)	3 (0.7)
Planctomycetota corrig.	3 (2.9)	20 (5.8)	10 (4.3)	27 (6.7)
Pseudomonadota corrig.	41 (40.2)	91 (26.2)	79 (33.8)	114 (28.4)
Thermodesulfobacteriota corrig.	0 (0.0)	0 (0.0)	0 (0.0)	1 (0.2)
Verrucomicrobiota corrig.	2 (2.0)	23 (6.6)	16 (6.8)	25 (6.2)
NA	0 (0.0)	2 (0.6)	1 (0.4)	0 (0.0)
Total	102 (100.0)	352 (100.0)	234 (100.0)	402 (100.0)

Table S8. Core node sequence variants with multiple links in the four sample groups.

Top 10% and 5% of total eukaryotic and prokaryotic node sequence variants (nSVs) were selected as core nSVs by link numbers and indicated along with their link numbers with their to indicated phyla (upper table: eukaryotic core nSVs; lower table: prokaryotic core nSVs). NA: Unassigned phylum. Genera from Blastn hits with the lowest e-values are indicated in parentheses. Yellow-colored cells: core nSVs shared by the four sample groups. Orange- and pale green-colored cells: core nSVs shared by the maize- and cabbage-cultivated soils, respectively. Red-colored nSVs: core nSVs shared by the field soils. Numbers in brackets: Total number of nSVs in the sample group.

Eukaryotic core nSVs

Field_1_maize (62)			Field_1_cabbage (121)			Field_2_maize (84)			Field_2_cabbage (170)		
**nSV**	**Links**	**Phylum**	**nSV**	**Links**	**Phylum**	**nSV**	**Links**	**Phylum**	**nSV**	**Links**	**Phylum (blastn closest genus)**
SV_28	10	Cercozoa	SV_100	20	Ascomycota	SV_2	29	Basidiomycota	SV_231	32	NA (genus *Pompholyxophrys*)
SV_4	9	Basidiomycota	SV_39	19	Ascomycota	SV_28	29	Cercozoa	SV_144	26	Cercozoa
SV_2	8	Basidiomycota	SV_5	18	Ascomycota	SV_41	27	Mucoromycota	SV_169	26	NA (genus *Pompholyxophrys*)
SV_7	6	Mucoromycota	SV_4	17	Basidiomycota	SV_8	25	Ascomycota	SV_25	25	Mucoromycota
SV_10	6	Mucoromycota	SV_43	17	Cercozoa	SV_84	23	Basidiomycota	SV_69	25	Cercozoa
SV_82	6	Basidiomycota	SV_63	17	Ciliophora	SV_4	22	Basidiomycota	SV_2	24	Basidiomycota
SV_84	6	Basidiomycota	SV_99	16	Cercozoa	SV_14	22	Ascomycota	SV_43	24	Cercozoa
			SV_15	15	Ascomycota	SV_30	21	Mucoromycota	SV_90	24	Cercozoa
			SV_87	15	Ascomycota	SV_32	20	Ascomycota	SV_115	24	NA(genus *Ochromonas*)
			SV_74	14	Ascomycota	SV_64	20	Oomycota	SV_28	23	Cercozoa
			SV_23	12	Ascomycota				SV_57	23	Cercozoa
			SV_32	12	Ascomycota				SV_124	23	Nematoda
			SV_113	12	Cercozoa				SV_4	22	Basidiomycota
									SV_51	21	Cercozoa
									SV_63	21	Ciliophora
									SV_13	20	Cercozoa
									SV_248	20	Nematoda

Prokaryotic core nSVs

Field_1_maize (102)			Field_1_cabbage (352)			Field_2_maize (234)			Field_2_cabbage (402)		
**nSV**	**Links**	**Phylum**	**nSV**	**Links**	**Phylum**	**nSV**	**Links**	**Phylum**	**nSV**	**Links**	**Phylum**
SV_228	9	Actinomycetota corrig.	SV_312	68	Pseudomonadota corrig.	SV_5	66	Pseudomonadota corrig.	SV_23	86	Pseudomonadota corrig.
SV_8	8	Pseudomonadota corrig.	SV_63	64	Pseudomonadota corrig.	SV_1	59	Pseudomonadota corrig.	SV_128	82	Pseudomonadota corrig.
SV_21	7	Acidobacteriota corrig.	SV_12	61	Verrucomicrobiota corrig.	SV_148	53	Pseudomonadota corrig.	SV_21	80	Acidobacteriota corrig.
SV_705	7	Pseudomonadota corrig.	SV_156	58	Acidobacteriota corrig.	SV_61	50	Pseudomonadota corrig.	SV_88	78	Acidobacteriota corrig.
			SV_300	57	Pseudomonadota corrig.	SV_23	49	Pseudomonadota corrig.	SV_285	76	Planctomycetota corrig.
			SV_136	55	Actinomycetota corrig.	SV_68	49	Gemmatimonadota corrig.	SV_32	75	Armatimonadota corrig.
			SV_143	55	Gemmatimonadota corrig.	SV_15	42	Acidobacteriota corrig.	SV_189	74	Gemmatimonadota corrig.
			SV_75	50	Planctomycetota corrig.	SV_7	40	Pseudomonadota corrig.	SV_389	71	Bacteroidota corrig.
			SV_104	50	Pseudomonadota corrig.	SV_22	40	Pseudomonadota corrig.	SV_42	69	Acidobacteriota corrig.
			SV_197	49	Acidobacteriota corrig.	SV_13	38	Verrucomicrobiota corrig.	SV_114	66	Pseudomonadota corrig.
			SV_40	48	Bacillota corrig.	SV_49	38	Pseudomonadota corrig.	SV_186	65	Planctomycetota corrig.
			SV_129	47	Acidobacteriota corrig.	SV_84	38	Verrucomicrobiota corrig.	SV_46	64	Gemmatimonadota corrig.
			SV_150	46	Planctomycetota corrig.	SV_88	38	Acidobacteriota corrig.	SV_305	63	Pseudomonadota corrig.
			SV_560	46	Gemmatimonadota corrig.				SV_164	62	Pseudomonadota corrig.
			SV_43	45	Actinomycetota corrig.				SV_66	61	Pseudomonadota corrig.
			SV_199	44	Acidobacteriota corrig.				SV_253	61	Candidatus Latescibacteria
			SV_264	44	Actinomycetota corrig.				SV_532	61	Acidobacteriota corrig.
			SV_14	43	Actinomycetota corrig.				SV_85	60	Chloroflexota corrig.
									SV_177	59	Pseudomonadota corrig.
									SV_243	59	Acidobacteriota corrig.

Table S9. Numbers and percentage of the core node sequence variants in each phylum.

The number of core node sequence variants (nSVs) derived from eukaryotic and prokaryotic phyla (upper and lower tables, respectively) and prokaryotic (bottom tables) phyla are shown for each sample group with the percentage of the total core nSVs indicated in parenthesis. Data in yellow-colored cells are referred to in the text.

Eukaryotic phyla	Field_1_maize	Field_1_cabbage	Field_2_maize	Field_2_cabbage
Ascomycota	0 (0.0)	8 (61.5)	3 (30.0)	0 (0.0)
Basidiomycota	4 (57.1)	1 (7.7)	3 (30.0)	2 (11.8)
Mucoromycota	2 (28.6)	0 (0.0)	2 (20.0)	1 (5.9)
Cercozoa	1 (14.3)	3 (23.1)	1 (10.0)	8 (47.1)
Ciliophora	0 (0.0)	1 (7.7)	0 (0.0)	1 (5.9)
Oomycota	0 (0.0)	0 (0.0)	1 (10.0)	0 (0.0)
Nematoda	0 (0.0)	0 (0.0)	0 (0.0)	2 (11.8)
NA	0 (0.0)	0 (0.0)	0 (0.0)	3 (17.6)
Total	7 (100.0)	13 (100.0)	10 (100.0)	17 (100.0)

Prokaryotic phyla	Field_1_maize	Field_1_cabbage	Field_2_maize	Field_2_cabbage
Acidobacteriota corrig.	1 (25.0)	4 (22.2)	2 (15.4)	5 (25.0)
Actinomycetota corrig.	1 (25.0)	4 (22.2)	0 (0.0)	0 (0.0)
Armatimonadota corrig.	0 (0.0)	0 (0.0)	0 (0.0)	1 (5.0)
Bacillota corrig.	0 (0.0)	1 (5.6)	0 (0.0)	0 (0.0)
Bacteroidota corrig.	0 (0.0)	0 (0.0)	0 (0.0)	1 (5.0)
Candidatus Latescibacteria	0 (0.0)	1 (5.6)	0 (0.0)	1 (5.0)
Chloroflexota corrig.	0 (0.0)	0 (0.0)	0 (0.0)	1 (5.0)
Gemmatimonadota corrig.	0 (0.0)	2 (11.1)	1 (7.7)	2 (10.0)
Planctomycetota corrig.	0 (0.0)	1 (5.6)	0 (0.0)	2 (10.0)
Pseudomonadota corrig.	2 (50.0)	4 (22.2)	8 (61.5)	7 (35.0)
Verrucomicrobiota corrig.	0 (0.0)	1 (5.6)	2 (15.4)	0 (0.0)
Total	4 (100.0)	18 (100.0)	13 (100.0)	20 (100.0)

Table S10. Crop- and field-specific eukaryotic sequence variants and associated taxa.

The following tables show the common node sequence variants (nSVs) shared by the four sample groups, nSVs specifically detected in maize- and cabbage-cultivated soils, and nSVs exclusively detected in field_1 and field_2 with their phyla and genera. These genera were assigned based on the top hits by Blastn searching. Cluster numbers of nSVs in each sample (e.g., F1_m: maze-cultivated field_1 samples) are indicated (see Supplementary Figs. S6 and S7). The core nSVs in each sample are also checked in the corresponding cells. Phyla in pale green and yellow cells are classified to fungi and protists, respectively. NA: Not assigned. The nSVs in yellow-colored cells are referred to in the text.

Common nSV	Phylum	Genus	Cluster No.				Core nSVs			
			F1_m	F1_c	F2_m	F2_c	F1_m	F1_c	F2_m	F2_c
SV_65	Ascomycota	*Brachyconidiellopsis*	2	1	1	1				
SV_42	Ascomycota	*Chaetomium*	2	1	1	1				
SV_5	Ascomycota	*Fusarium*	1	1	1	1		✓		
SV_11	Ascomycota	*Nais*	2	1	1	1				
SV_36	Ascomycota	*Saccobolus*	2	1	1	1				
SV_8	Ascomycota	*Trichocladium*	2	1	1	1			✓	
SV_4	Basidiomycota	*Solicoccozyma*	1	1	1	1	✓	✓	✓	✓
SV_2	Basidiomycota	*Tausonia*	1	1	1	1	✓		✓	✓
SV_43	Cercozoa	*Heteromita*	8	1	2	1		✓		✓
SV_28	Cercozoa	*Spongomonas*	1	1	1	1	✓		✓	✓
SV_13	Cercozoa	*Trinema*	1	1	1	1				✓
SV_63	Ciliophora	*Colpoda*	1	1	1	1		✓		✓
SV_7	Mucoromycota	*Mortierella, Linnemannia, Podila*	1	1	1	11	✓			
**Maize-specific nSV**	**Phylum**	**Genus**								
SV_37	Ascomycota	*Plectosphaerella*	1		1					
SV_94	Ascomycota	*Stemphylium*	7		1					
SV_84	Basidiomycota	*Cystofilobasidium*	1		1		✓		✓	
SV_203	Cercozoa	*Neocercomonas*	5		1					
**Cabbage-specific nSV**	**Phylum (Clade)**	**Genus**								
SV_52	Ascomycota	*Lepidosphaeria, Asteromassaria*		1		1				
SV_93	Ascomycota	*Arthrobotrys, Gamsylella, Dactylellina, Orbilia*		3		1				
SV_87	Ascomycota	*Aspergillus*		1		1		✓		
SV_133	Cercozoa	*Eocercomonas*		16		2				
SV_223	Cercozoa	*Eocercomonas/Protaspis*		1		2				
SV_53	Cercozoa	*Euglypha*		1		1				
SV_137	Cercozoa	*Euglypha*		7		2				
SV_135	Cercozoa	*Kraken*		5		1				
SV_227	Cercozoa	*Nudifila, Protaspis*		18		8				
SV_175	Cercozoa	*Rhogostoma*		5		1				
SV_104	Cercozoa	*Trinema*		1		1				
SV_121	Chytridiomycota	*Spizellomyces*		1		2				
SV_243	Ciliophora	*Colpoda*		17		1				
SV_347	NA	*Dimorpha*		1		24				
SV_129	NA	NA		8		1				
SV_311	Ochrophyta	*Paraphysomonas*		3		9				
SV_216	Ochrophyta	*Spumella*		3		2				
SV_167	Tubulinea	*Vermamoeba*		1		1				
**Field_1-specific nSV**	**Phylum**	**Genus**								
SV_45	Ascomycota	*Alternaria*	1	9						
SV_91	Basidiomycota	*Minimedusa*	1	6						
SV_82	Basidiomycota	*Saitozyma, Vanrija*	1	1			✓			
SV_99	Cercozoa	*Spongomonas*	8	1				✓		
SV_123	Ciliophora	*Colpoda*	4	1						
SV_114	Endomyxa	*Theratromyxa, Arachnula*	1	1						
SV_79	Nematoda	*Diploscapter*	9	4						
SV_49	Oomycota	*Globisporangium*	1	3						
SV_111	Tubulinea	*Vermamoeba, Hartmannella*	1	6						
**Field_2-specific nSV**	**Phylum**	**Genus**								
SV_16	Ascomycota	*Microthecium*			1	1				
SV_127	Ascomycota	*Chalazion*			1	1				
SV_112	Ascomycota	*Colletotrichum*			1	16				
SV_73	Ascomycota	*Lasiobolus*			1	1				
SV_44	Ascomycota	*Mariannaea*			1	1				
SV_96	Ascomycota	*Miladina, Scutellinia*			1	1				
SV_207	Basidiomycota	*Apiotrichum*			1	1				
SV_90	Cercozoa	*Bodomorpha*			1	1				✓
SV_196	Cercozoa	*Cercomonas*			1	1				
SV_146	Cercozoa	*Protaspis*			2	6				
SV_92	Cercozoa	*Rhogostoma*			1	1				
SV_193	Cercozoa	*Trinema*			1	3				
SV_97	Endomyxa	*Plasmodiophora*			1	1				
SV_142	Endomyxa	*Spongospora*			1	1				
SV_25	Mucoromycota	*Actinomortierella*			1	1				✓
SV_30	Mucoromycota	*Mortierella, Linnemannia, Podila*			1	1			✓	
SV_41	Mucoromycota	*Mortierella, Linnemannia, Podila*			1	11			✓	
SV_107	NA	*Spumella*			1	1				

Table S11. Crop- and field-specific prokaryotic node sequence variants and associated taxa.

See the legend for Supplementary Table S10. Taxa in yellow-colored cells are referred to in the text.

Common nSV	Phylum	Genus	Cluster No.				Core nSVs			
			F1_m	F1_c	F2_m	F2_c	F1_m	F1_c	F2_m	F2_c
SV_43	Actinomycetota corrig.	*Gaiella*	3	1	1	1		✓		
SV_86	Bacillota corrig.	*Neobacillus*	3	1	1	1				
SV_45	Pseudomonadota corrig.	*Methyloceanibacter*	13	1	1	1				
SV_16	Pseudomonadota corrig.	*Povalibacter*	1	1	1	1				
SV_23	Pseudomonadota corrig.	*Rhodoplanes*	13	1	1	1			✓	✓
SV_67	Pseudomonadota corrig.	*Skermanella*	7	1	1	4				
SV_1	Pseudomonadota corrig.	*Sphingomonas*	12	1	1	1			✓	

Maize-specific nSV	Phylum	Genus	F1_m	F1_c	F2_m	F2_c	F1_m	F1_c	F2_m	F2_c
SV_437	Actinomycetota corrig.	*Embleya*	4		9					
SV_121	Actinomycetota corrig.	*Nocardioides*	7		1					
SV_117	Bacteroidota corrig.	*Ginsengibacter*	3		1					
SV_159	Gemmatimonadota corrig.	*Gemmatimonas*	6		1					
SV_8	Pseudomonadota corrig.	*Rhodanobacter*	3		1		✓			
SV_24	Pseudomonadota corrig.	*Rhodanobacter*	3		1					
SV_432	Pseudomonadota corrig.	*Sphingomonas*	2		1					
SV_448	Verrucomicrobiota corrig.	*Lacunisphaera*	8		1					

Cabbage-specific nSV	Phylum	Genus	F1_m	F1_c	F2_m	F2_c	F1_m	F1_c	F2_m	F2_c
SV_129	Acidobacteriota corrig.	*Candidatus Koribacter*		1		1		✓		
SV_42	Acidobacteriota corrig.	*Chloracidobacterium*		1		1				✓
SV_124	Acidobacteriota corrig.	*Chloracidobacterium*		1		1				
SV_316	Acidobacteriota corrig.	*Chloracidobacterium*		1		1				
SV_11	Acidobacteriota corrig.	*Luteitalea*		1		1				
SV_120	Acidobacteriota corrig.	*Luteitalea*		1		1				
SV_242	Acidobacteriota corrig.	*Luteitalea*		1		1				
SV_243	Acidobacteriota corrig.	*Luteitalea*		1		1				✓
SV_600	Acidobacteriota corrig.	*Luteitalea*		1		9				
SV_238	Acidobacteriota corrig.	*Paludibaculum*		1		1				
SV_395	Acidobacteriota corrig.	*Paludibaculum*		1		12				
SV_595	Acidobacteriota corrig.	*Paludibaculum*		6		1				
SV_326	Actinomycetota corrig.	*Actinomarinicola*		17		2				
SV_72	Actinomycetota corrig.	*Baekduia*		1		1				
SV_64	Actinomycetota corrig.	*Gaiella*		1		3				
SV_161	Actinomycetota corrig.	*Gaiella*		1		12				
SV_103	Actinomycetota corrig.	*Geodermatophilus*		1		7				
SV_146	Actinomycetota corrig.	*Rhabdothermincola*		1		1				
SV_383	Actinomycetota corrig.	*Rhabdothermincola*		3		1				
SV_404	Actinomycetota corrig.	*Rhabdothermincola*		2		1				
SV_56	Actinomycetota corrig.	*Streptomyces*		1		1				
SV_169	Bacteroidota corrig.	*Flavisolibacter*		1		1				
SV_70	Gemmatimonadota corrig.	*Gemmatimonas*		1		1				
SV_93	Gemmatimonadota corrig.	*Gemmatimonas*		1		1				
SV_132	Gemmatimonadota corrig.	*Gemmatirosa*		1		1				
SV_443	Gemmatimonadota corrig.	*Gemmatirosa*		1		2				
SV_690	Planctomycetota corrig.	*Aureliella*		22		6				
SV_82	Planctomycetota corrig.	*Humisphaera*		15		1				
SV_107	Planctomycetota corrig.	*Humisphaera*		1		1				
SV_186	Planctomycetota corrig.	*Humisphaera*		15		1				✓
SV_217	Planctomycetota corrig.	*Humisphaera*		1		1				
SV_347	Pseudomonadota corrig.	*Acidibrevibacterium*		2		12				
SV_216	Pseudomonadota corrig.	*Azorhizobium, Methylocapsa*		3		1				
SV_402	Pseudomonadota corrig.	*Azospirillum*		1		9				
SV_153	Pseudomonadota corrig.	*Chondromyces*		1		1				
SV_232	Pseudomonadota corrig.	*Dokdonella*		1		1				
SV_51	Pseudomonadota corrig.	*Dongia*		1		1				
SV_145	Pseudomonadota corrig.	*Dongia*		1		5				
SV_470	Pseudomonadota corrig.	*Hyphomicrobium*		3		1				
SV_292	Pseudomonadota corrig.	*Lysobacter*		2		1				
SV_102	Pseudomonadota corrig.	*Nitrosovibrio*		1		1				
SV_31	Pseudomonadota corrig.	*Novimethylophilus, Azoarcus, Methyloversatilis*		1		1				
SV_223	Pseudomonadota corrig.	*Povalibacter*		1		1				
SV_460	Pseudomonadota corrig.	*Povalibacter*		1		1				
SV_110	Pseudomonadota corrig.	*Rhizomicrobium*		1		1				
SV_340	Pseudomonadota corrig.	*Rhodoplanes*		1		1				
SV_83	Pseudomonadota corrig.	*Rudaea*		1		1				
SV_293	Pseudomonadota corrig.	*Steroidobacter*		1		1				
SV_187	Pseudomonadota corrig.	*Trinickia*		16		1				
SV_77	Pseudomonadota corrig.	*Usitatibacter*		1		1				
SV_44	Pseudomonadota corrig.	*Vulgatibacter*		1		1				
SV_37	Verrucomicrobiota corrig.	*Pedosphaera*		1		1				

Field_1-specific nSV	Phylum	Genus	F1_m	F1_c	F2_m	F2_c	F1_m	F1_c	F2_m	F2_c
SV_231	Acidobacteriota corrig.	*Luteitalea*	6	4						
SV_381	Actinomycetota corrig.	*Conexibacter*	2	18						
SV_274	Actinomycetota corrig.	*Gaiella*	5	1						
SV_614	Actinomycetota corrig.	*Gaiella*	10	3						

SV_228	Actinomycetota corrig.	*Ilumatobacter*	2	4			✓			
SV_575	Actinomycetota corrig.	*Solirubrobacter*	18	1						
SV_201	Actinomycetota corrig.	*Streptomyces*	4	5						
SV_494	Bacteroidota corrig.	*Parafilimonas*	3	1						
SV_476	Chloroflexota corrig.	*Brevefilum*	15	4						
SV_523	Chloroflexota corrig.	*Dehalogenimonas*	16	8						
SV_143	Gemmatimonadota corrig.	*Gemmatirosa*	5	1				✓		
SV_259	Pseudomonadota corrig.	*Dongia*	16	1						
SV_692	Pseudomonadota corrig.	*Halochromatium, Inmirania*	14	23						
SV_250	Pseudomonadota corrig.	*Povalibacter*	6	1						
SV_298	Verrucomicrobiota corrig.	*Pedosphaera*	8	4						

**Field_2-specific nSV**	**Phylum**	**Genus**								
SV_203	Acidobacteriota corrig.	*Chloracidobacterium*			1	3				
SV_342	Acidobacteriota corrig.	*Chloracidobacterium*			1	1				
SV_396	Acidobacteriota corrig.	*Luteitalea*			1	1				
SV_414	Acidobacteriota corrig.	*Luteitalea*			1	1				
SV_556	Acidobacteriota corrig.	*Luteitalea*			1	1				
SV_154	Acidobacteriota corrig.	*Paludibaculum*			1	1				
SV_15	Acidobacteriota corrig.	*Pyrinomonas*			1	1			✓	
SV_171	Acidobacteriota corrig.	*Pyrinomonas*			1	1				
SV_221	Acidobacteriota corrig.	*Pyrinomonas*			1	1				
SV_229	Acidobacteriota corrig.	*Pyrinomonas*			1	1				
SV_268	Acidobacteriota corrig.	*Pyrinomonas*			1	1				
SV_455	Actinomycetota corrig.	*Intrasporangium*			6	6				
SV_441	Actinomycetota corrig.	*Rhabdothermincola*			1	1				
SV_653	Actinomycetota corrig.	*Rhabdothermincola, Actinomarinicola*			1	1				
SV_622	Actinomycetota corrig.	*Virgisporangium*			1	2				
SV_32	Armatimonadota corrig.	*Fimbriimonas*			1	1				✓
SV_283	Bacteroidota corrig.	*Chitinophaga*			1	8				
SV_251	Bacteroidota corrig.	*Flavisolibacter*			1	1				
SV_275	Bacteroidota corrig.	*Ginsengibacter*			1	1				
SV_155	Bacteroidota corrig.	*Paraflavitalea*			1	1				
SV_417	Bacteroidota corrig.	*Pseudoflavitalea, Chitinophaga*			1	1				
SV_240	Chloroflexota corrig.	*Sphaerobacter*			9	5				
SV_139	Chloroflexota corrig.	*Tepidiforma*			1	1				
SV_170	Chloroflexota corrig.	*Tepidiforma*			1	1				
SV_196	Chloroflexota corrig.	*Tepidiforma*			1	1				
SV_3	Bacillota corrig.	*Peribacillus*			1	1				
SV_189	Gemmatimonadota corrig.	*Gemmatimonas*			1	1				✓
SV_310	Gemmatimonadota corrig.	*Gemmatimonas*			1	1				
SV_74	Gemmatimonadota corrig.	*Gemmatirosa*			1	1				
SV_76	Gemmatimonadota corrig.	*Gemmatirosa*			1	1				
SV_33	Nitrospirota corrig.	*Nitrospira*			1	1				
SV_105	Nitrospirota corrig.	*Nitrospira*			1	1				
SV_406	Planctomycetota corrig.	*Humisphaera*			1	1				
SV_480	Planctomycetota corrig.	*Humisphaera*			1	1				
SV_625	Planctomycetota corrig.	*Humisphaera*			1	1				
SV_475	Pseudomonadota corrig.	*Anaeromyxobacter*			1	1				
SV_304	Pseudomonadota corrig.	*Arenimonas*			1	1				
SV_617	Pseudomonadota corrig.	*Aurantiacibacter*			1	8				
SV_19	Pseudomonadota corrig.	*Azoarcus*			1	1				
SV_176	Pseudomonadota corrig.	*Azoarcus*			1	1				
SV_79	Pseudomonadota corrig.	*Erythrobacter*			3	1				
SV_640	Pseudomonadota corrig.	*Geoalkalibacter*			1	1				
SV_573	Pseudomonadota corrig.	*Labilithrix*			3	9				
SV_140	Pseudomonadota corrig.	*Nitrosospira*			1	1				
SV_142	Pseudomonadota corrig.	*Nitrosovibrio*			1	1				
SV_279	Pseudomonadota corrig.	*Nitrosovibrio*			1	1				
SV_248	Pseudomonadota corrig.	*Nitrospirillum*			1	1				
SV_561	Pseudomonadota corrig.	*Polyangium*			4	1				
SV_81	Pseudomonadota corrig.	*Povalibacter*			6	1				
SV_502	Pseudomonadota corrig.	*Povalibacter*			1	4				
SV_345	Pseudomonadota corrig.	*Robbsia*			4	1				
SV_29	Pseudomonadota corrig.	*Sphingomonas*			1	1				
SV_61	Pseudomonadota corrig.	*Sphingomonas*			1	1			✓	
SV_73	Pseudomonadota corrig.	*Sphingomonas*			1	1				
SV_78	Pseudomonadota corrig.	*Sphingomonas*			1	1				
SV_112	Pseudomonadota corrig.	*Sphingomonas*			1	1				
SV_157	Pseudomonadota corrig.	*Sphingomonas*			1	1				
SV_258	Pseudomonadota corrig.	*Sphingomonas*			1	1				
SV_225	Pseudomonadota corrig.	*Tepidimonas*			1	5				
SV_677	Pseudomonadota corrig.	*Vulgatibacter*			1	1				
SV_34	Verrucomicrobiota corrig.	*Chthoniobacter*			1	1				
SV_206	Verrucomicrobiota corrig.	*Chthoniobacter*			1	1				
SV_263	Verrucomicrobiota corrig.	*Chthoniobacter*			8	1				
SV_290	Verrucomicrobiota corrig.	*Chthoniobacter*			1	1				
SV_141	Verrucomicrobiota corrig.	*Pedosphaera*			2	1				
SV_271	Verrucomicrobiota corrig.	*Pedosphaera*			1	1				
SV_795	Verrucomicrobiota corrig.	*Pedosphaera*			10	14				

Supplementary Figures

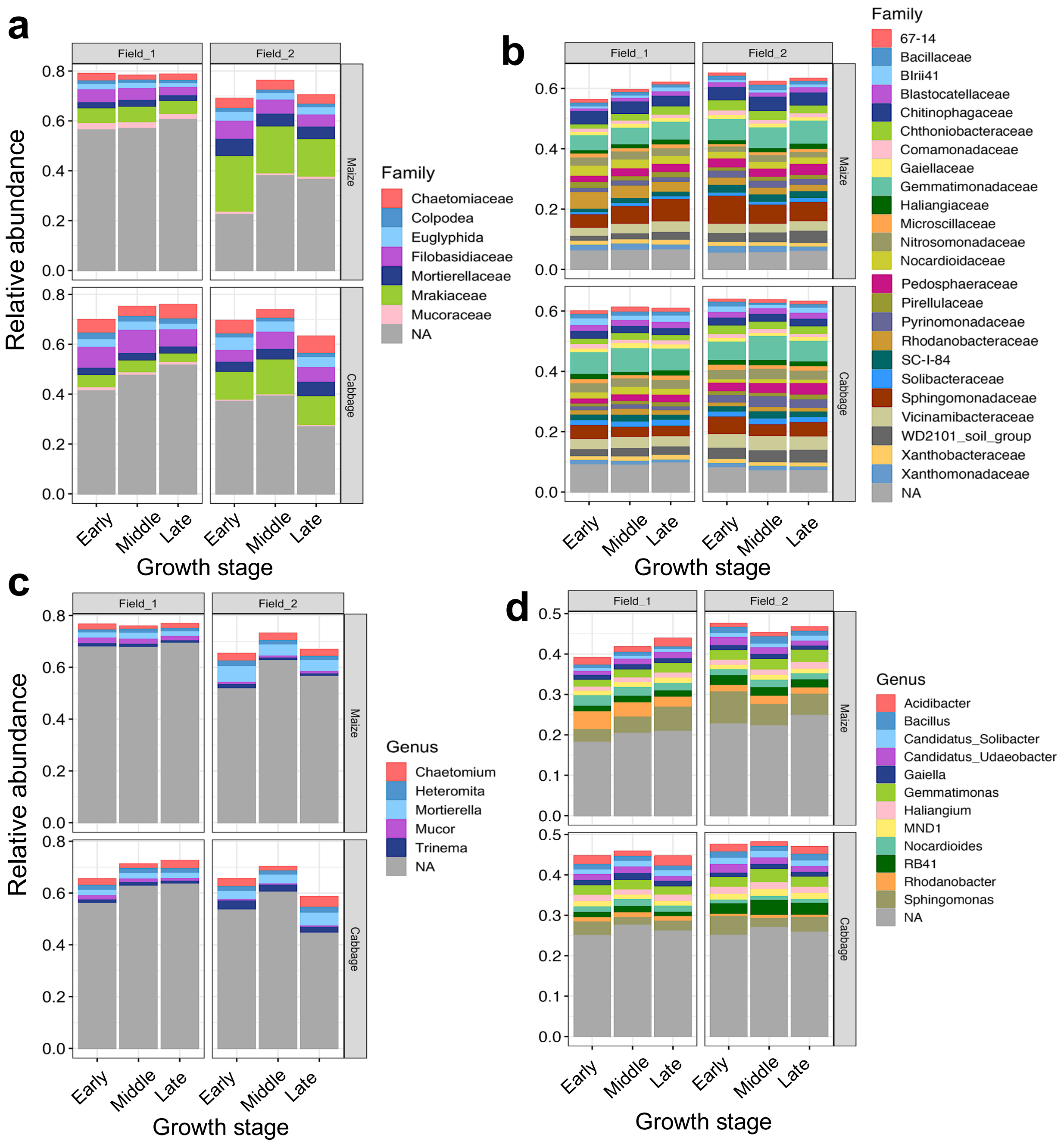

Supplementary Figure S1. Relative abundance of eukaryotic (a, c), prokaryotic (b, d) families (a, b), and genera (c, d) in the soils from two agricultural fields cultivating maize and cabbage at three different growth stages. Taxa are indicated by the colors shown on the right of the figures (note: some eukaryotic taxa are incorrectly classified in the SILVA database such as the order Euglyphida). Eukaryotic and prokaryotic sequence variants (SVs) with a relative abundance less than 0.5% and 1% were omitted. NA, not assigned family or genus.

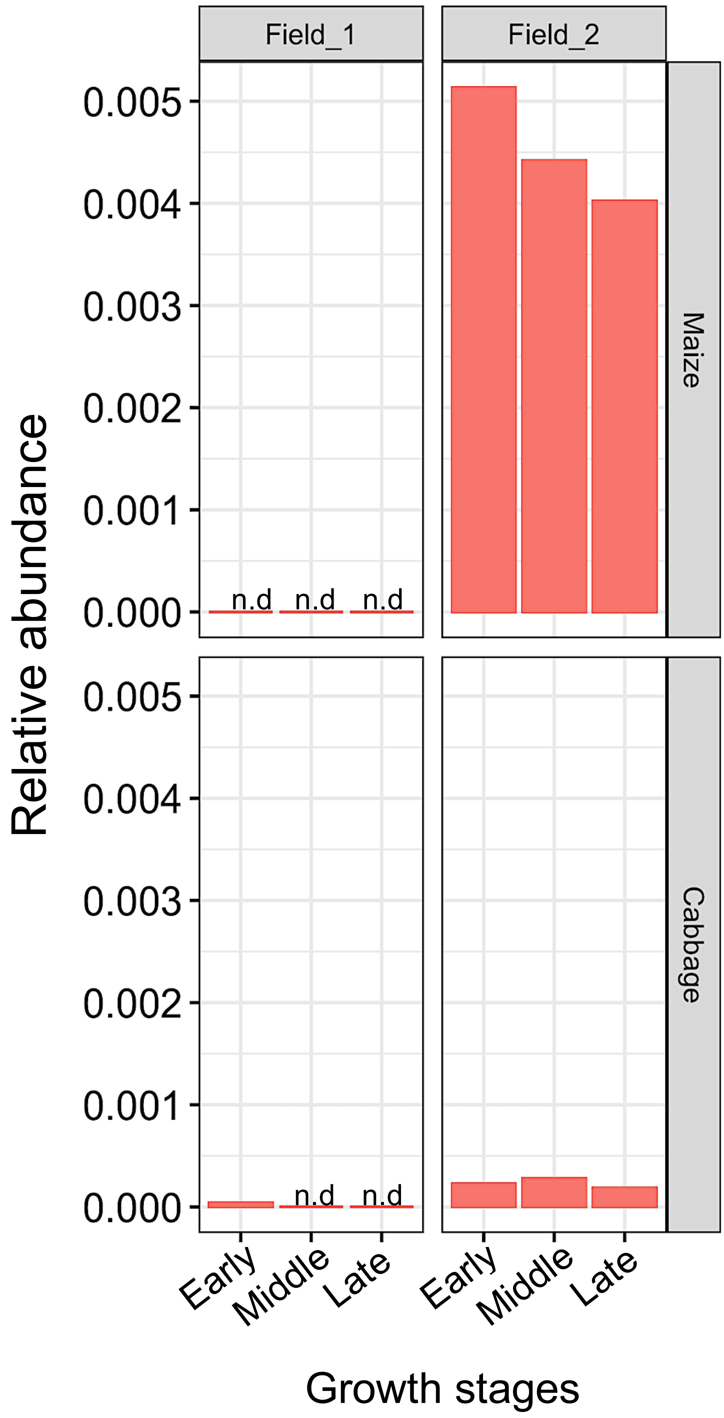

Supplementary Figure S2. Relative abundance of *Plasmodiophora brassicae*-derived sequence variant (SV_97) in total eukaryotic SVs. n.d.: not detected.

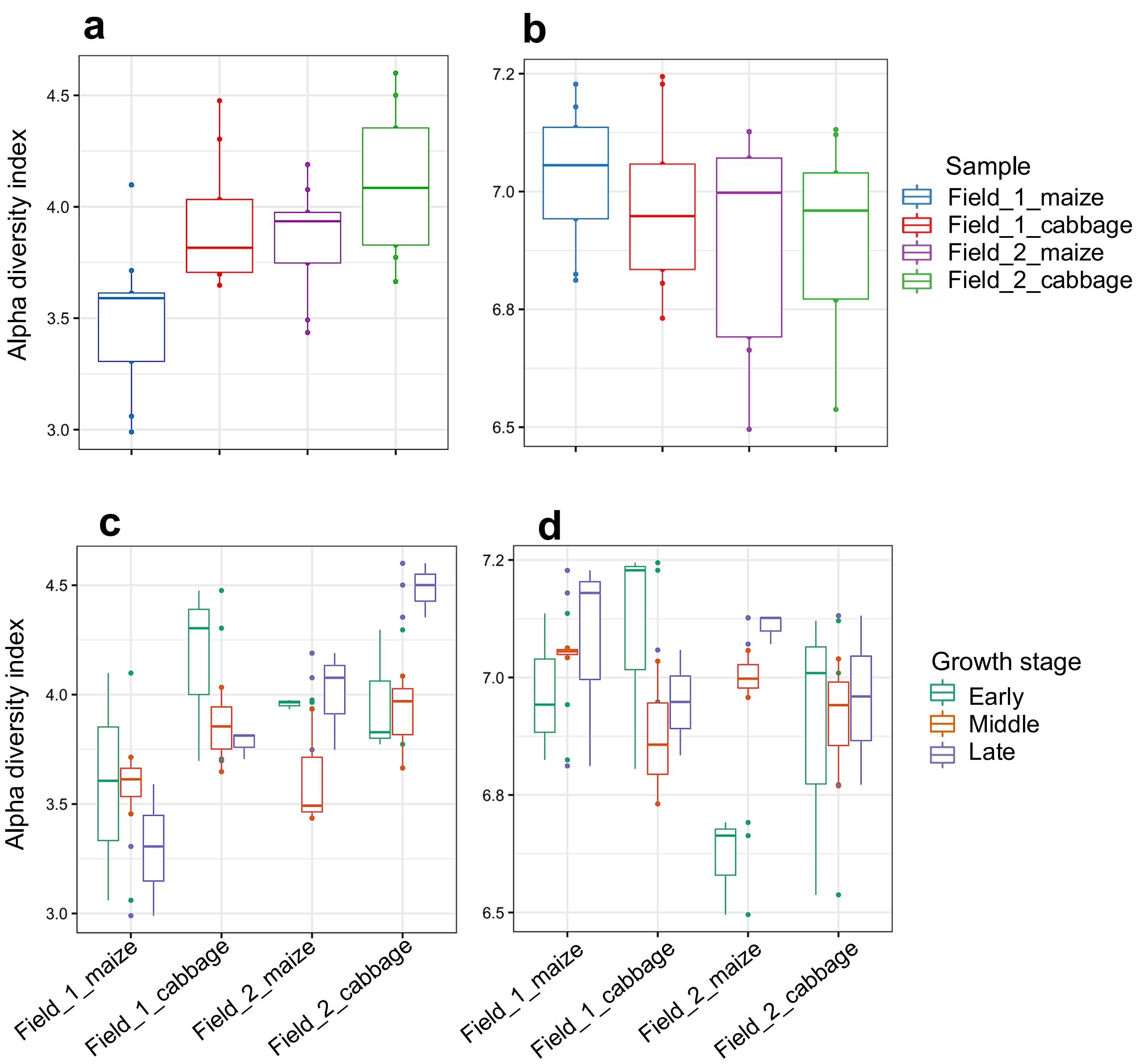

Supplementary Figure S3. Boxplots showing eukaryotic (a) and prokaryotic (b) α-diversity by Shannon index across the four sample groups from two different crops and fields. The α-diversities of eukaryotes (c) and prokaryotes (d) across the plant growth stages are indicated in each sample group by boxplots. Sample groups (a, b) and growth stages (c, d) are shown on the right.

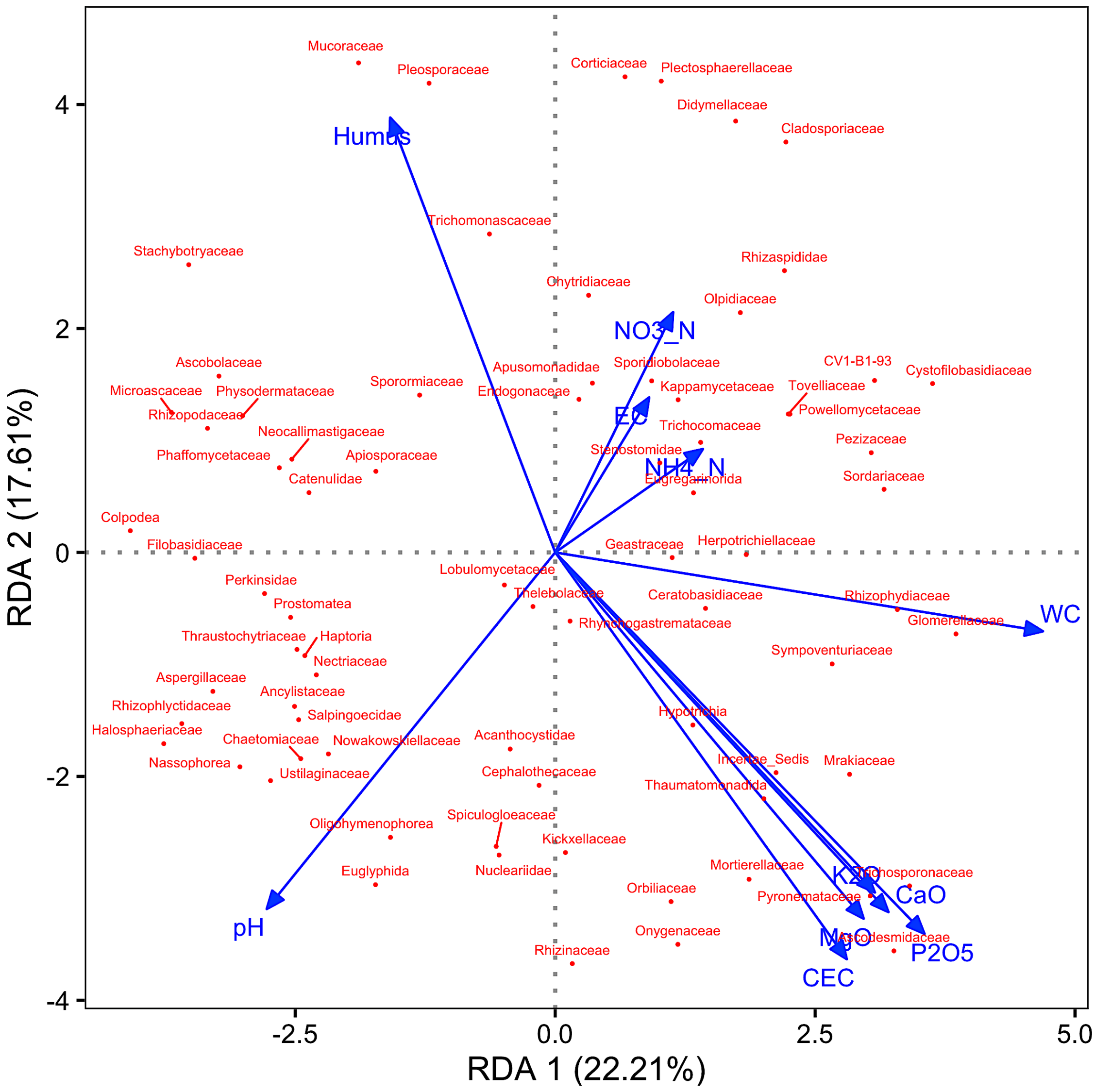

Supplementary Figure S4a. Redundancy analysis (RDA) of eukaryotic families in the samples showing soil chemical properties as vectors. Abbreviations of chemical properties: cation-exchange capacity (CEC), pH in water (pH), nitrate nitrogen (NO3_N), ammonium nitrogen (NH4_N), exchangeable potassium (K_2O), exchangeable magnesium (MgO), exchangeable calcium (CaO), electric conductivity (EC), available phosphorus (P_2O_5), humus content (Humus), and water content (WC).

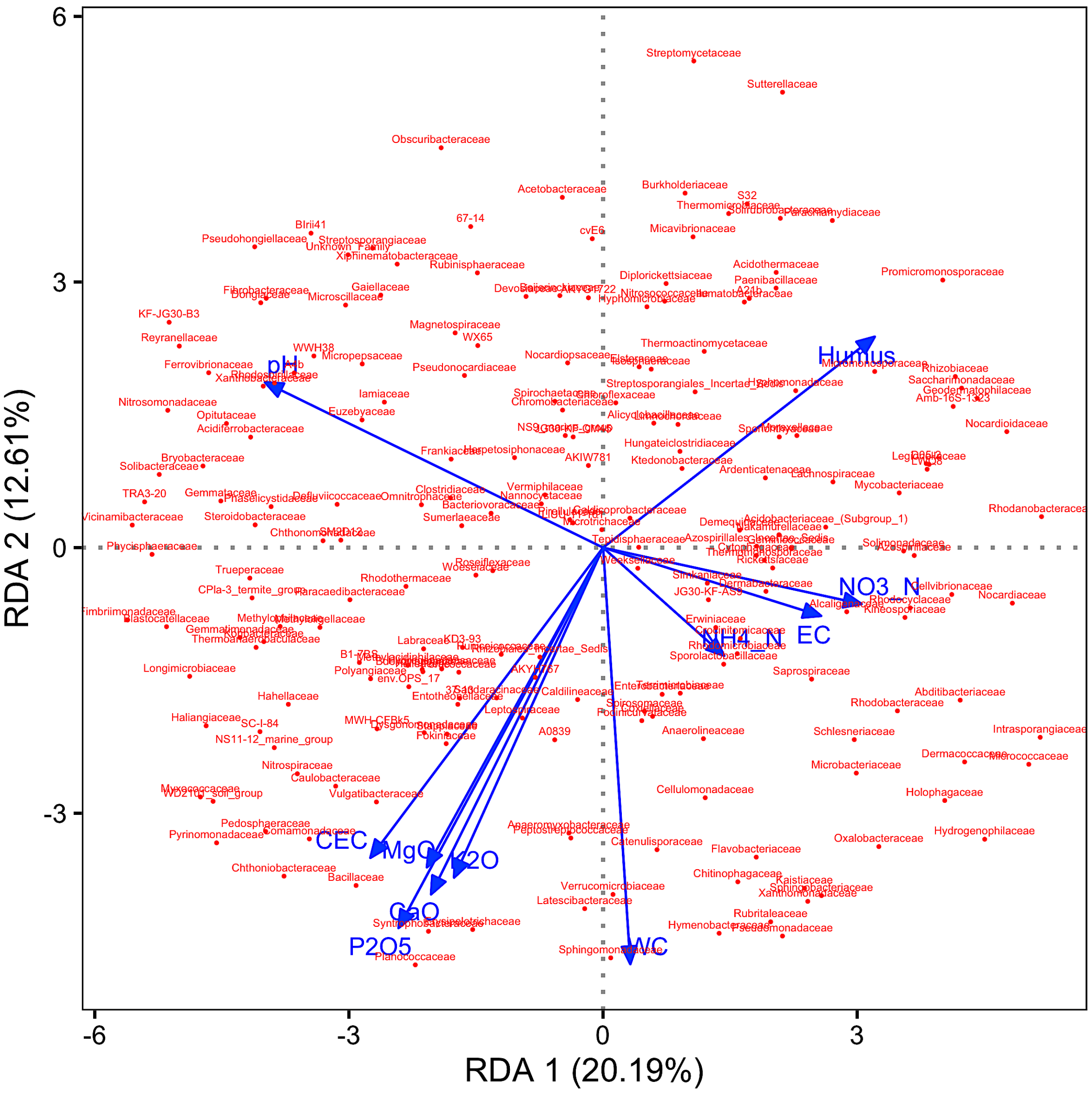

Supplementary Figure S4b. Redundancy analysis (RDA) of prokaryotic families in the samples showing soil chemical properties as vectors. The abbreviations of chemical properties are shown in the legend of Supplementary Figure S4a.

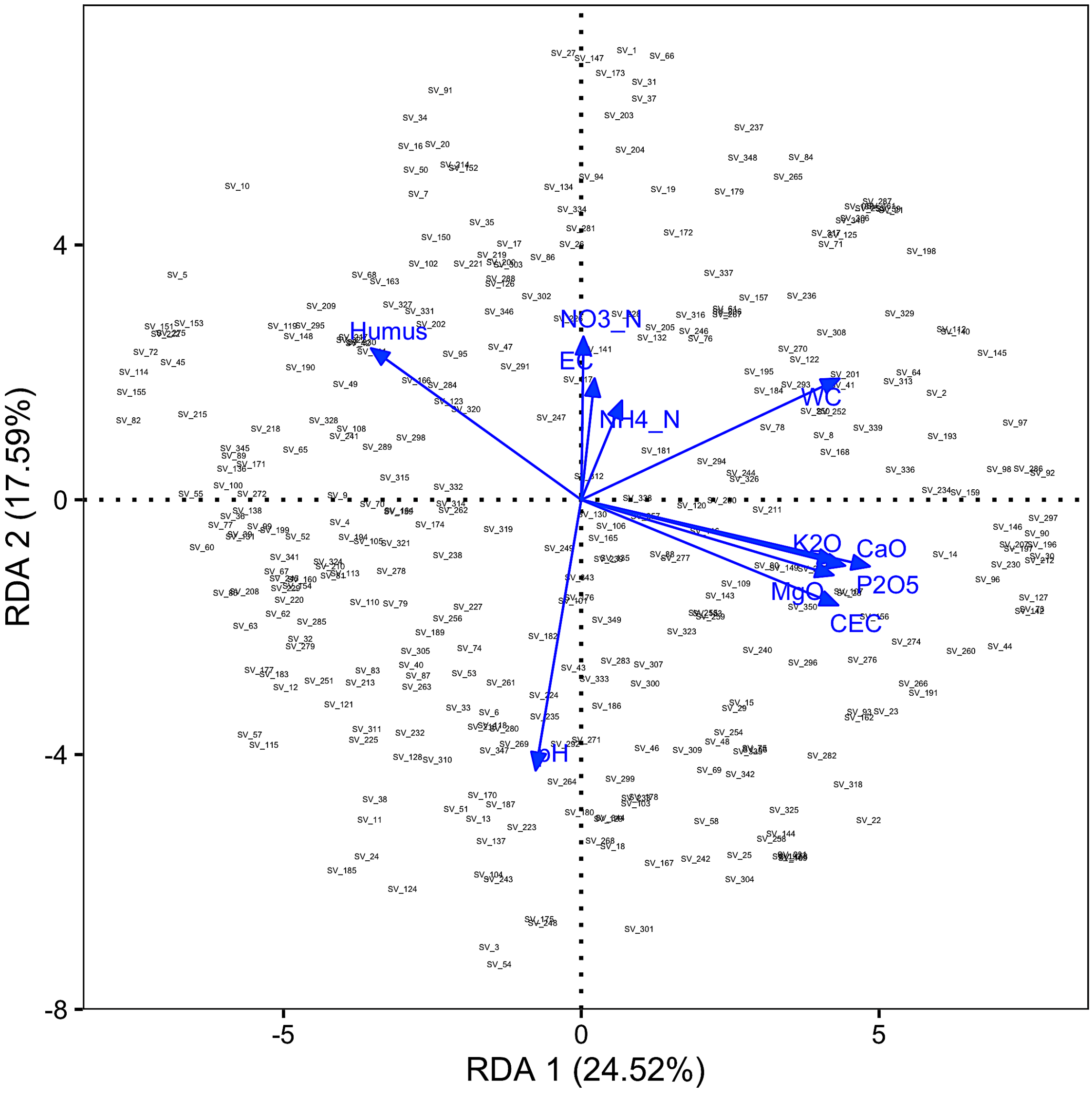

Supplementary Figure S4c. Redundancy analysis (RDA) of major eukaryotic sequence variants (SVs) in the samples showing soil chemical properties as vectors. The 350 most abundant SVs were subjected to RDA. The abbreviations of chemical properties are shown in the legend of Supplementary Figure S4a.

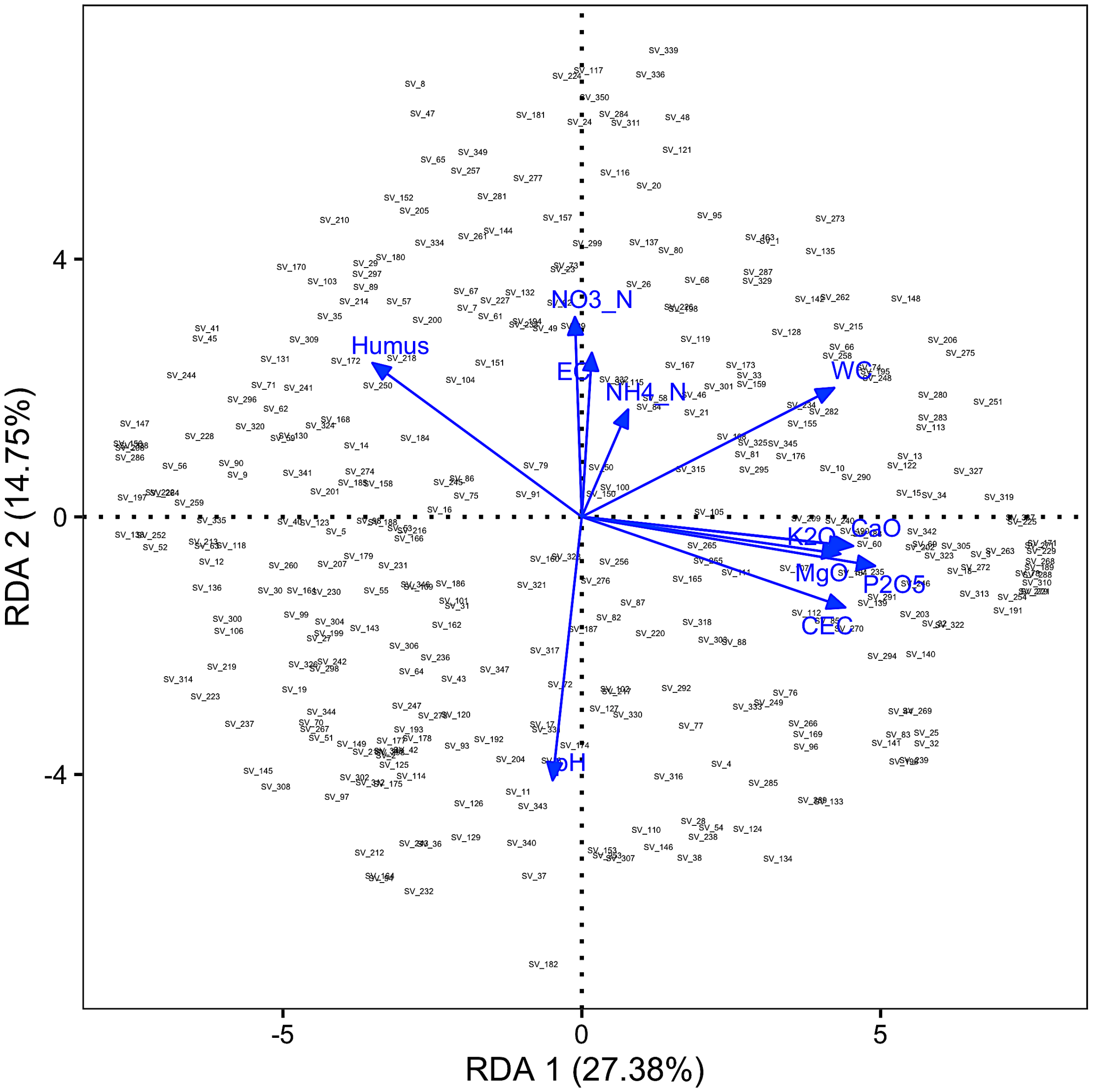

Supplementary Figure S4d. Redundancy analysis (RDA) of major prokaryotic sequence variants (SVs) in the samples showing soil chemical properties as vectors. The 350 most abundant SVs were subjected to RDA. The abbreviations of chemical properties are shown in the legend of Supplementary Figure S4a.

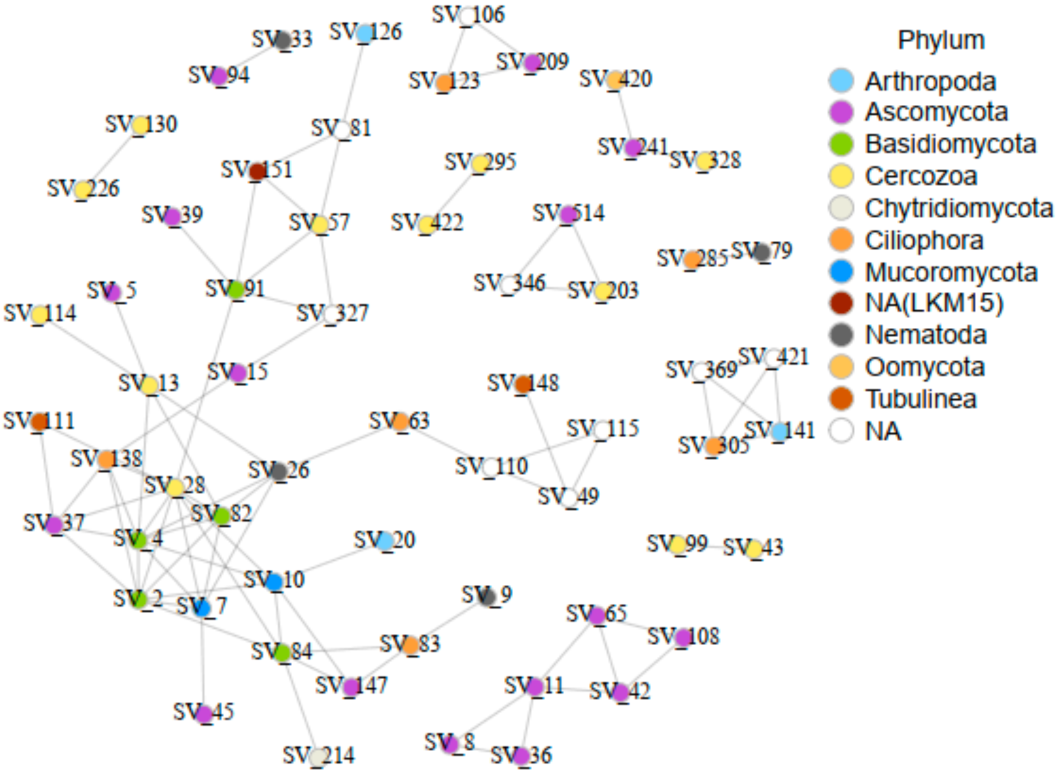

Supplementary Figure 5a. Network of eukaryotic node sequence variants (nSVs) in the maize-cultivated field_1 soils. The names of nSVs and links are shown, and the phyla of nSVs are indicated by color.

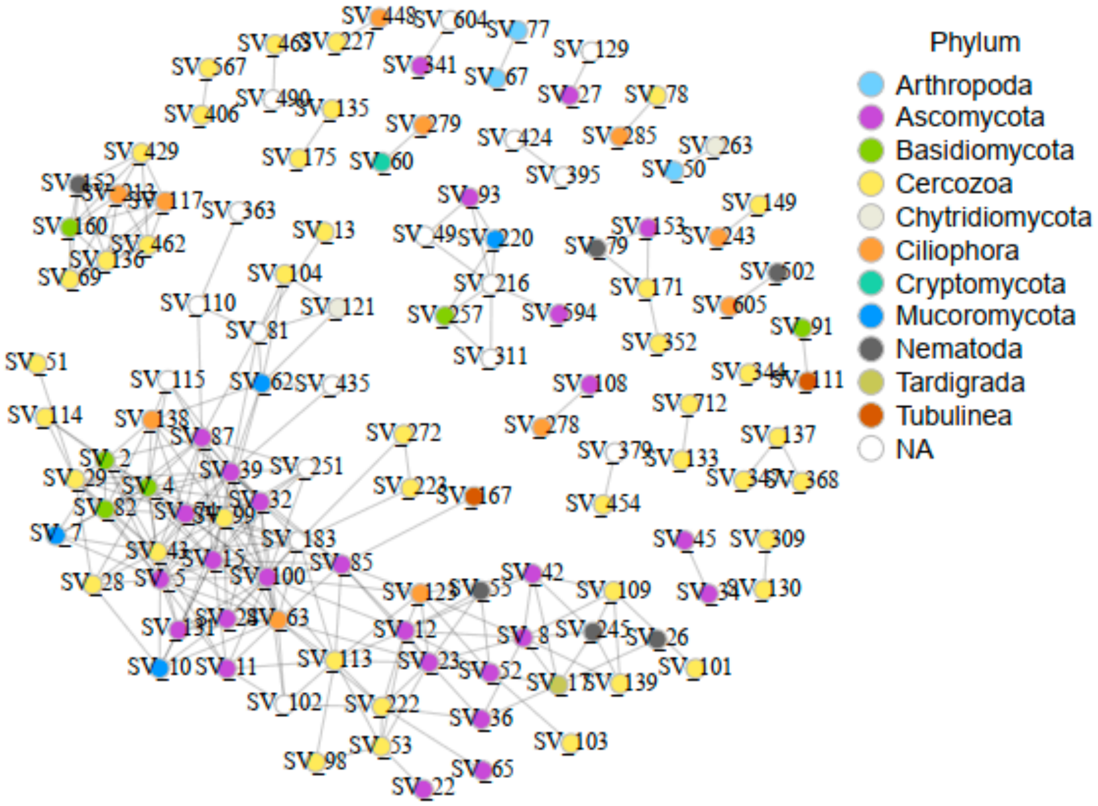

Supplementary Figure 5b. Network of eukaryotic node sequence variants (nSVs) in the cabbage-cultivated field_1 soils. The names of nSVs and links are shown, and the phyla of nSVs are indicated by color.

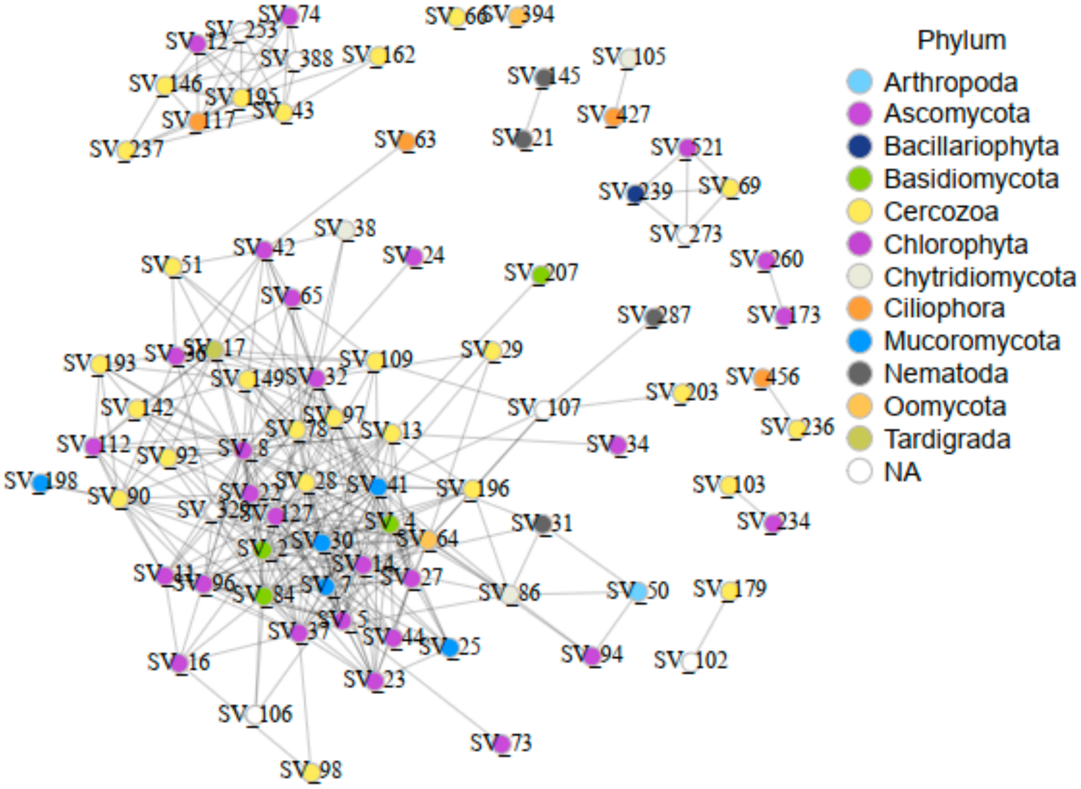

Supplementary Figure 5c. Network of eukaryotic node sequence variants (nSVs) in the maize-cultivated field_2 soils. The names of nSVs and links are shown, and the phyla of nSVs are indicated by color.

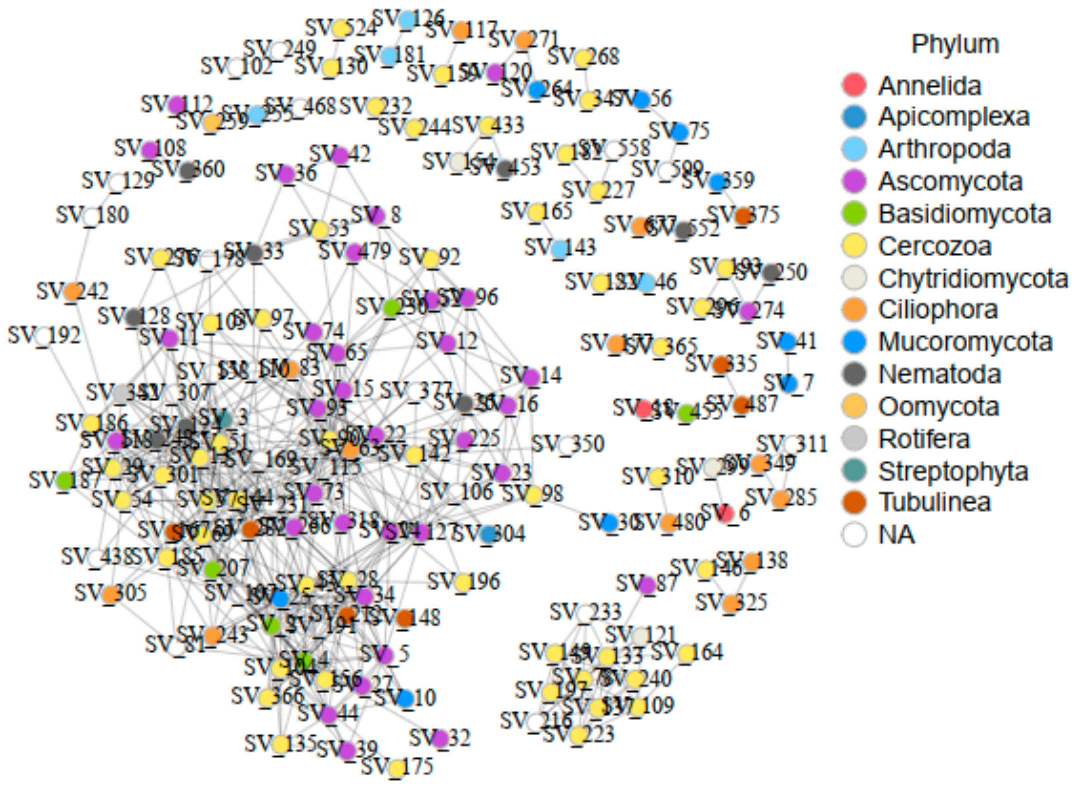

Supplementary Figure 5d. Network of eukaryotic node sequence variants (nSVs) in the cabbage-cultivated field_2 soils. The names of nSVs and links are shown, and the phyla of nSVs are indicated by color.

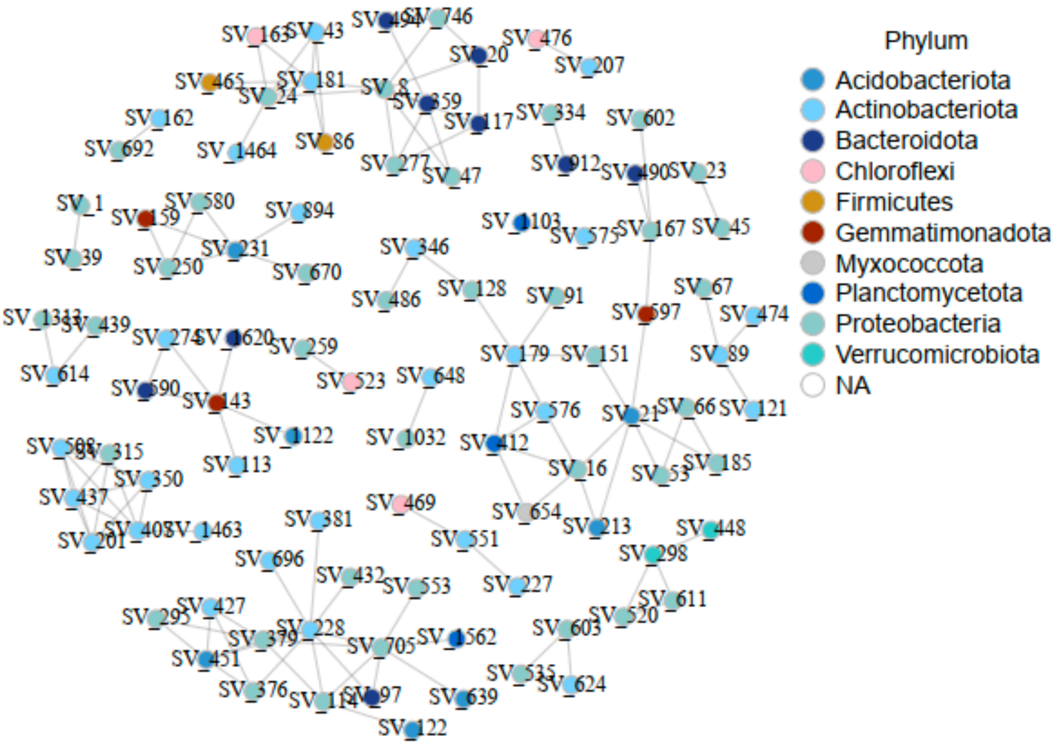

Supplementary Figure 5e. Network of prokaryotic node sequence variants (nSVs) in the maize-cultivated field_1 soils. The names of nSVs and links are shown, and the phyla of nSVs are indicated by color.

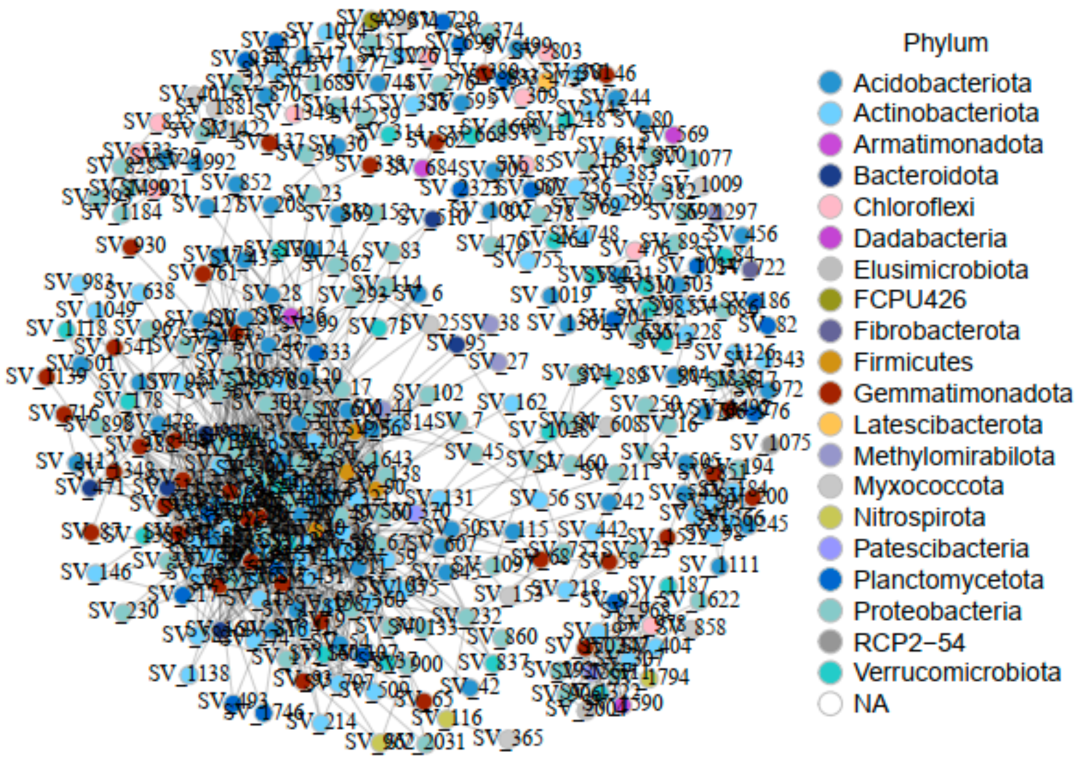

Supplementary Figure 5f. Network of prokaryotic node sequence variants (nSVs) in the cabbage-cultivated field_1 soils. The names of nSVs and links are shown, and the phyla of nSVs are indicated by color. NA: phylum not assigned.

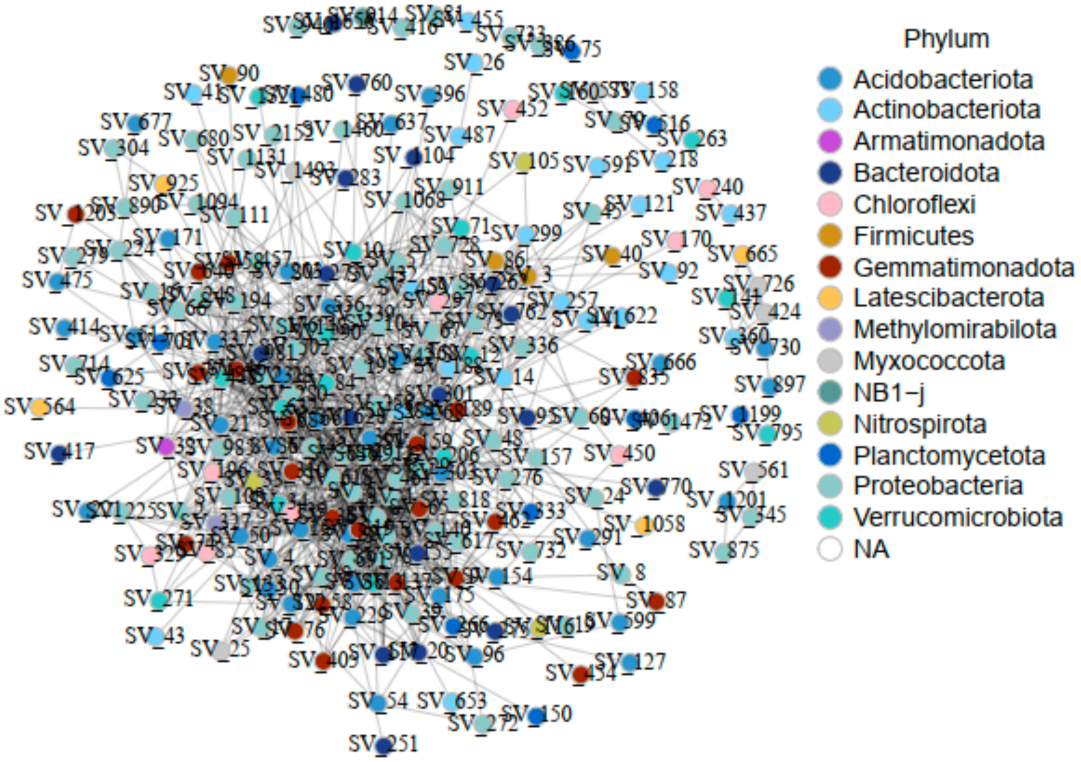

Supplementary Figure 5g. Network of prokaryotic node sequence variants (nSVs) in the maize-cultivated field_2 soils. The names of nSVs and links are shown, and the phyla of nSVs are indicated by color. NA: phylum not assigned.

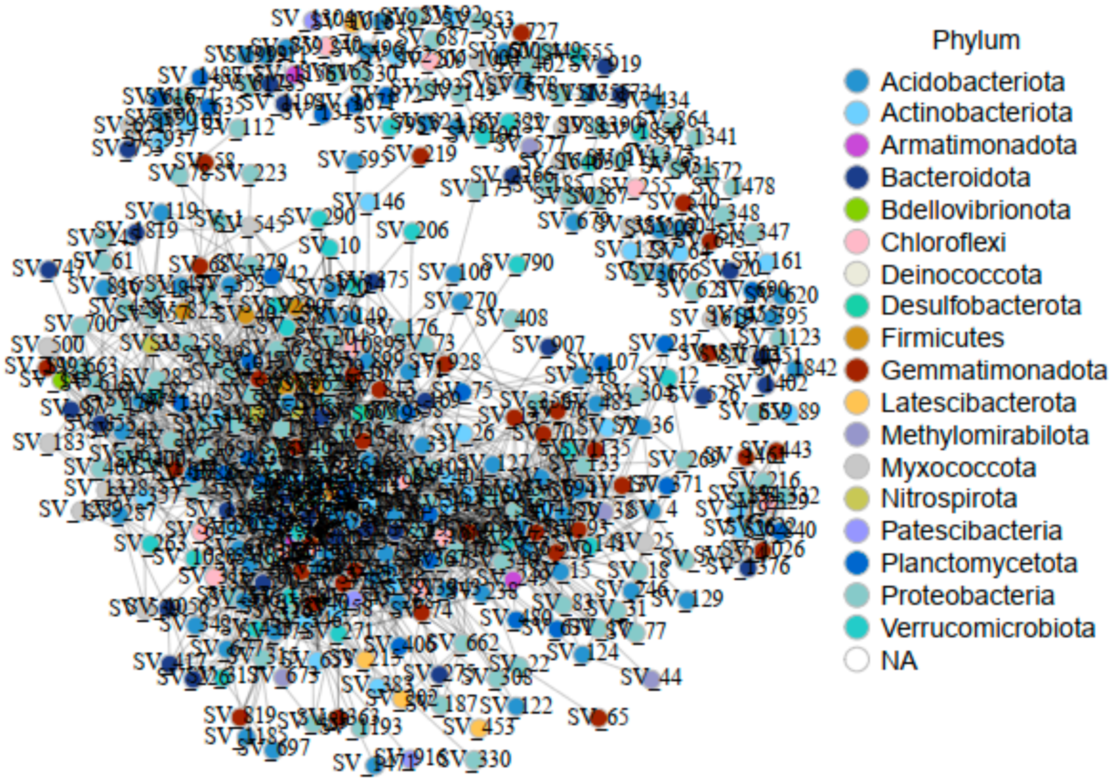

Supplementary Figure 5h. Network of prokaryotic node sequence variants (nSVs) in the cabbage-cultivated field_2 soils. The names of nSVs and links are shown, and the phyla of nSVs are indicated by color. NA: phylum not assigned.

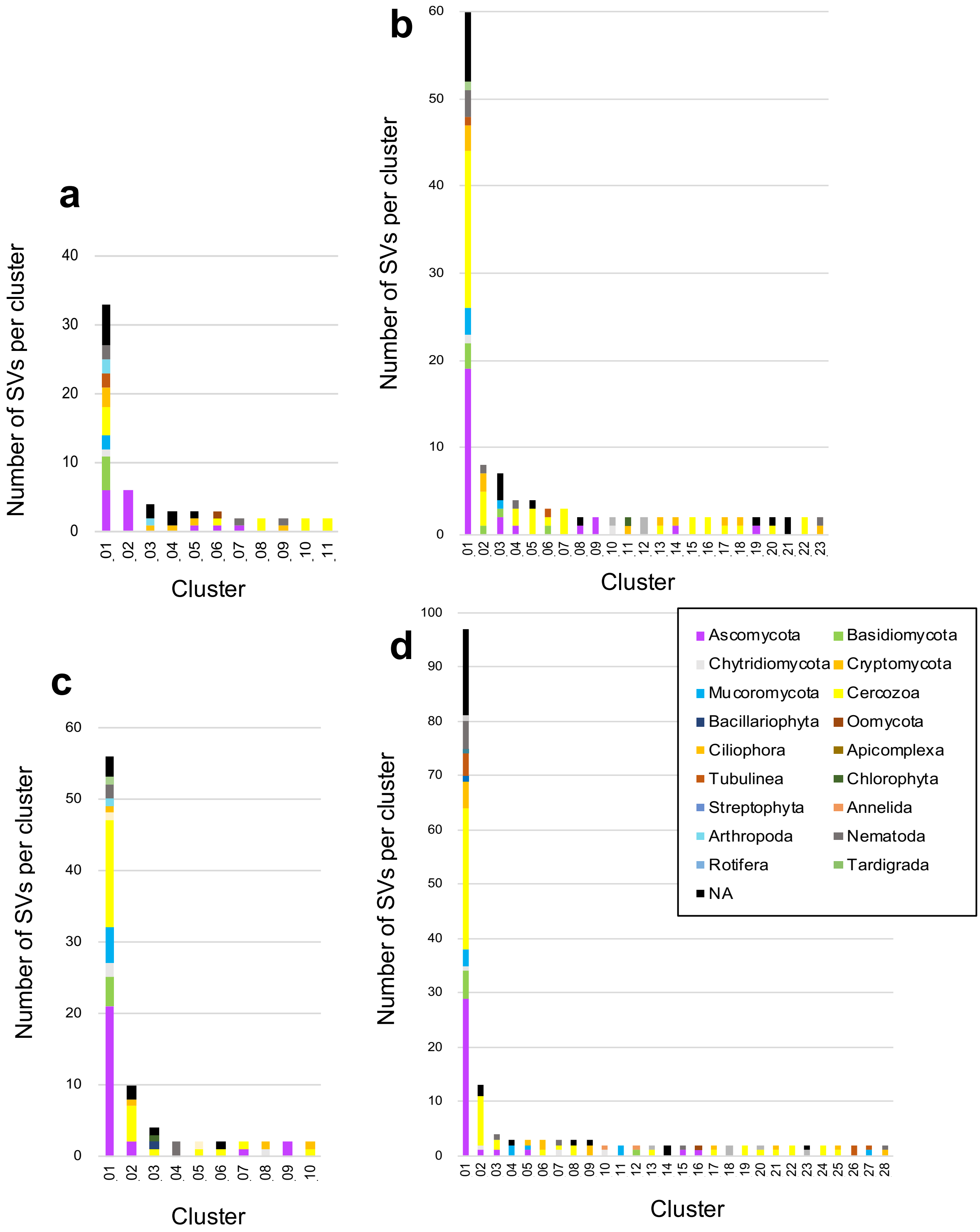

Supplementary Figure S6. Histograms of the numbers of eukaryotic node sequence variants (nSVs) per cluster in sample group networks (a, maize-cultivated field_1 soils; b, cabbage-cultivated field_1 soils; c, maize-cultivated field_2 soils; d, cabbage-cultivated field_2 soils). Phyla for nSVs are indicated by colors in the box. NA, not assigned phylum.

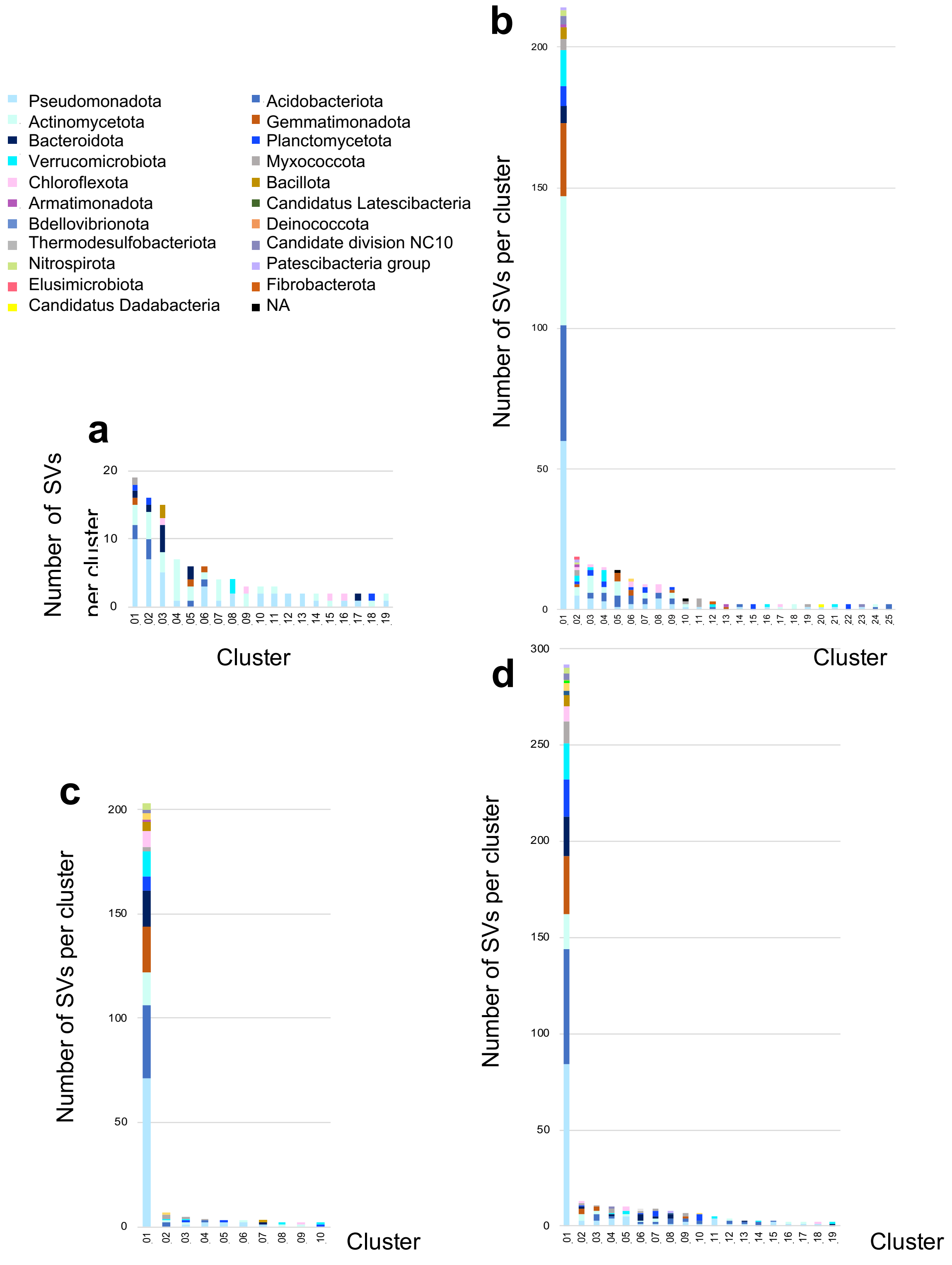

Supplementary Figure S7. Histograms of the numbers of prokaryotic node sequence variants (nSVs) per cluster in the network of the sample group (a, maize-cultivated field_1 soils; b, cabbage-cultivated field_1 soils; c, maize-cultivated field_2 soils; d, cabbage-cultivated field_2 soils). nSVs phyla are indicated by colors in the box. NA, not assigned phylum.
